# Focused screening of mitochondrial metabolism reveals a crucial role for a tumor suppressor Hbp1 in ovarian reserve

**DOI:** 10.1038/cdd.2016.47

**Published:** 2016-05-20

**Authors:** Z Dong, M Huang, Z Liu, P Xie, Y Dong, X Wu, Z Qu, B Shen, X Huang, T Zhang, J Li, J Liu, T Yanase, C Zhou, Y Xu

**Affiliations:** 1 MOE Key Laboratory of Model Animal for Disease Study, Model Animal Research Center, Nanjing University, Nanjing, China; 2 Cambridge-Suda Genomic Research Center, Soochow University, Suzhou, China; 3 State Key Laboratory of Reproductive Medicine, Nanjing Medical University, Nanjing, China; 4 Department of Endocrinology and Diabetes Mellitus, School of Medicine, Fukuoka University, Fukuoka, Japan; 5 Department of Biochemistry and Molecular Medicine and Comprehensive Cancer Center, Institute for Pediatric Regenerative Medicine, University of California Davis, School of Medicine, Sacramento, CA, USA

## Abstract

Granulosa cells (GCs) are tightly associated with fertility and the fate of ovarian follicles. Mitochondria are the central executers of apoptosis. However, the genetic basis underlying mitochondrial modulation in GCs during the ovarian development is poorly understood. Here, CRISPR/Cas9-mediated genetic screening was used to identify genes conferring mitochondrial metabolism in human GCs. The results uncovered roles for several tumor suppressors, including *HBP1*, in the augmentation of mitochondrial function. Focused analysis revealed that high-mobility group (HMG)-box transcription factor 1 (Hbp1) levels regulate mitochondrial biogenesis, which is associated with global changes in transcription including *Tfam*. The systemic or granulosa-specific but not oocyte-specific ablation of *Hbp1* promoted follicle growth and oocyte production, and is associated with the reduced apoptotic signals in mouse GCs. Consistent with increased mitochondrial function and attenuated GC apoptosis, the regulation of Hbp1 conferred substantial protection of ovarian reserve. Thus, the results of the present study provide a critical target to understand the control of the reproductive lifespan.

During ovarian folliculogenesis, recruited primordial follicles develop into antral follicles, which are characterized by the rapid proliferation and differentiation of GCs, and extensive oocyte enlargement.^1^ These dynamic processes require energy substrates that constitute a requisite gateway to the follicle development. Thus, mitochondrial adaptation is considered a fundamental process to match the energy demands during the ovarian cycle. However, little information is available regarding the regulation of mitochondrial function in GCs and the role for this process in follicle growth. This information is critical, as GC apoptosis has a key role in follicular atresia,^2^ and multiple apoptotic pathways emanate from the mitochondria.^3^ Thus, the regulation of mitochondrial function in GCs could represent a desirable target for promoting follicle growth, preventing ovarian aging and enhancing fertility therapy.

A breakthrough in understanding how primordial follicles are activated from the dormant pool was obtained from studies on phosphatase and tensin homolog(PTEN)^4^ and mammalian target of rapamycin complex 1/tuberous sclerosis proteins 1 and 2 (TSC1/2),^5^ demonstrating that the genetic ablation of *PTEN* or *TSC1/2* induces the excessive activation and depletion of the primordial follicle pool. *PTEN* and *TSC1/2* were initially identified as tumor suppressor genes,^6, 7^ suggesting that the primordial activation and growth of follicles share some common characters with tumor cells. These tumor suppressor genes might act as ‘brakes' to control primordial follicle initiation before oocyte maturation. The sensitivity of tumor suppressor genes to cellular energy status and defined roles in growth suggest that tumor suppressor genes affect mitochondrial function in GCs and thereby modulate follicle growth.

Thus, we established a stable screening system in human GC tumor-derived cells (KGN) using the CRISPR/Cas9 system and generated 24 tumor suppressor gene knockout collections. We next focused on functional studies of high-mobility group (HMG)-box transcription factor 1 (Hbp1), a member of the sequence-specific HMG family of transcription factors identified in this genetic screening, demonstrating that the genetic ablation of *Hbp1* in mice improved mitochondrial function in GCs, reduced caspase 3 activation and unexpectedly enhanced ovarian reserve. These primary negative regulators may be novel targets for modulating mitochondrial function to improve human fertility.

## Results

### Generation of loss-of-function collections of tumor suppressor genes in KGN cells

**Figure 1:**
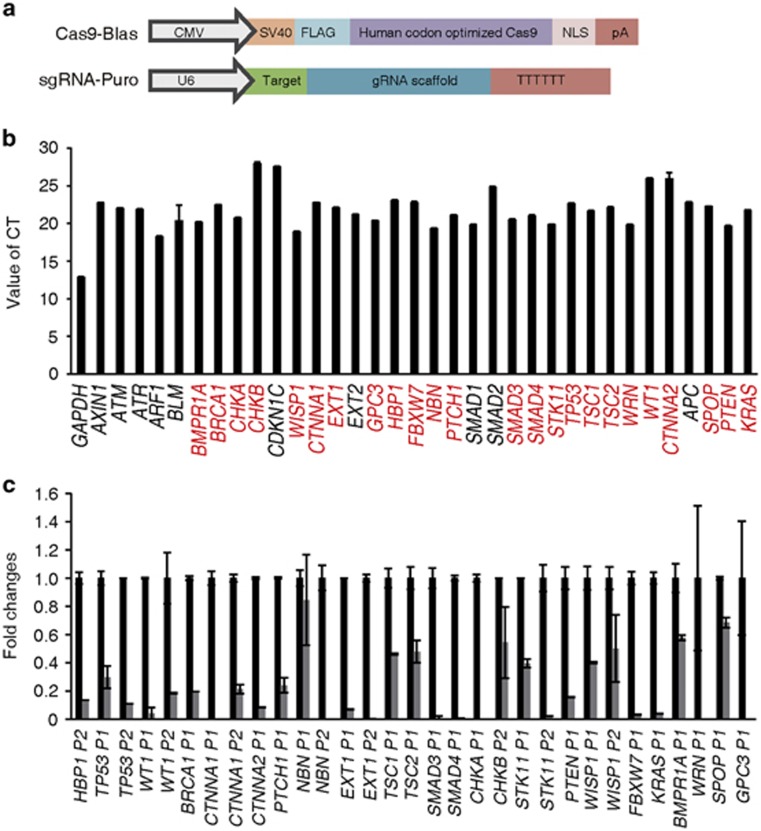
Efficient and rapid gene knockouts of enriched tumor suppressors in human KGN cells by CRISPR/Cas9 approach. (**a**) Design of the two vectors expressing Cas9 and sgRNA. Each vector contains a distinct selection marker to facilitate the co-selection of cells expressing the two vectors: expression vector for Cas9 with the blasticidin selection maker (Blast) and expression of sgRNA with the puromycin selection maker (Puro), respectively. (**b**) The mRNA levels of tumor suppressor genes expressed in KGN cells were determined through Q-PCR. The red letters represented the candidate genes for further analysis. The data are presented as the mean values±S.D. of three independent experiments. (**c**) Deletion efficacy of tumor suppressor genes by CRISPR/Cas9 was determined through real-time PCR at the targeted region. Primer1 means P1 (F1R1). Primer2 means P2 (F2R2). All of the results are represented as mean values±S.D. (*n*=3). The results are confirmed by three independent experiments

The CRISPR/CAS9 system was used to generate targeted mutations in tumor suppressor genes in KGN cells.^8^ To obtain cell lines expressing CAS9 and single guide RNA (sgRNA), we constructed two vectors (Figure 1a) with distinct antibiotic selection markers, in which *Cas9* expressed blasticidin and sgRNA expressed puromycin, as previously described.^9^ To increase the efficiency, two sgRNAs were designed to span the coding region within a distance of 100–400 bp, and genomic quantitative RT-PCR (Q-PCR) was employed to screen the efficacy of gene ablation through CRISPR/CAS9 in the following targeted KGN cell collections (Supplementary Figure 1a; Supplementary Methods and Materials). These approaches demonstrated successful gene knockout of enhanced green fluorescent protein (EGFP) in EGFP-expressing KGN cells (Supplementary Figure 1b–d).

To screen tumor suppressor genes in KGN cells, we overlapped three data sets from Gene Expression Omnibus, GSE 980760, GSE 980762 and GSE980765,^10^ acquired from KGN cells and data set of GSE20466^11^ that exhibited a >1.2-fold difference in the expression level in response to pregnant mare's serum gonadotropin (PMSG) treatment with the tumor suppressor gene database (http://bioinfo.mc.vanderbilt.edu/TSGene). A total of 34 genes from 418 common genes that expressed in the KGN cells, including *HBP1*, *PTEN*, *TP53* and *TSC2*, were selected for this pilot study. We also examined the expression of these genes in the KGN cells by Q-PCR (Figure 1b). To efficiently disrupt these genes, we designed multiple pairs of sgRNA, and only those that achieved sufficient gene knockout (24 genes, labeled as red letters in Figure 1b) were selected to establish cell strains for further functional analysis (Figure 1c).

### Functional screening of mitochondrial metabolism affected by loss-of-function of tumor suppressor genes

**Figure 2:**
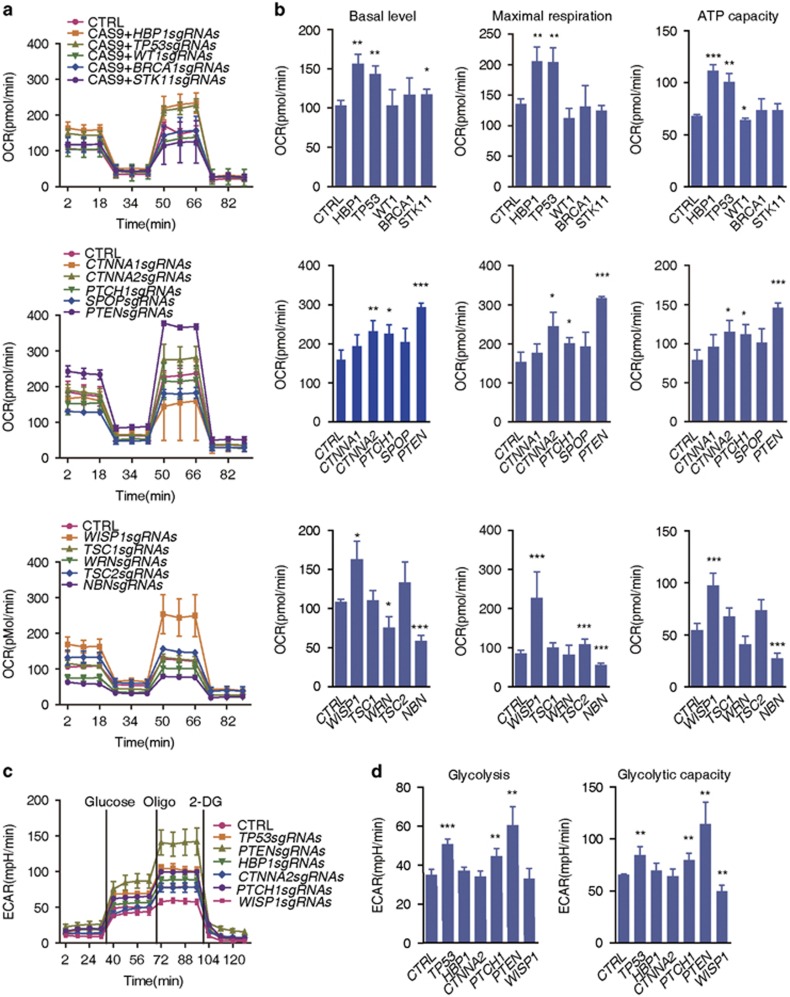
Dynamic changes of mitochondrial bioenergetics after deletion of various tumor suppressor genes in human KGN Cells. (**a**) OCR levels were monitored using the Seahorse Bioscience Extra Cellular Flux Analyzer in real time. The Cell numbers were counted with Moxi Z cell counter (ORFLO, Ketchum, ID, USA), then 50 000 cells were seeded onto 24-well plates for XF24 in 250 μl of growth medium and incubated with 5% CO_2_ for 6–7 h (thereafter). The cells were sequentially treated as indicated with oligomycin (olig, 1 μM), *p*-trifluoromethoxy carbonyl cyanide phenyl hydrazone (FCCP, 0.5 μM), antimycin A (1 μM) and rotenone (rote, 1 μM). OCR, indicative of OXPHOS. Vertical lines indicate the time points for the administration of the corresponding inhibitors. All data were further normalized to the ratios of the indicated genotype to control (genomic ß-actin level using q-PCR) (thereafter) and were presented as the mean values±S.D., *n*=3 per group in the Seahorse experiments. (**b**) Histograms of the basal respiration, maximal respiration and the ATP capacity of KGN cells deficient in various tumor suppressor genes compared with the normal controls. The data are presented as the mean values±S.D., *n*=3 per group in the Seahorse experiments. *P<0.05, **P<0.01 and ***P<0.001. (**c**) ECAR levels were monitored using the Seahorse Bioscience Extra Cellular Flux Analyzer in real time in KGN cells. The cells were treated sequentially with glucose (100 mM), oligomycin (oligo, 1 μM) and 2-deoxyglucose (2-DG, 100 mM). (**d**) Basal levels and glycolytic capacity of ECAR were determined in KGN cells with different genotypes. Each column represents the mean values±S.D., *n*=3 per group in the Seahorse experiments

To assess the impact of loss-of-function of tumor suppressor genes on mitochondrial function in the targeted KGN cells, cellular oxidative phosphorylation (OXPHOS) was monitored using a Seahorse Bioscience Extracellular Flux Analyzer (North Billerica, MA, USA) based on the measurements of the oxygen consumption rate (OCR). To avoid off-targeting effects or insufficient knockout, at least two KGN cell strains retaining consistent results among different colonies were labeled as candidate target genes. Most of the candidate target genes had no effect on mitochondrial respiration (Supplementary Table S1; Supplementary Figure 2). We identified five target genes in KGN cells, *HBP1*, *TP53*, *PTEN, CTNNA2* and *WISP1*. These genes displayed a significant increase in OCR of basal, maximal respiration and adenosine triphosphate (ATP) production capacity (Figures 2a and b; Supplementary Table S1). Among these genes, *TP53*, *PTEN* and *WISP1* have been associated with mitochondrial function in other types of cells,^12, 13, 14, 15, 16^ whereas an association of *HBP1* and *CTNNA2* with the regulation of mitochondrial function is demonstrated for the first time in the present study.

Activated oncogenes or deficient tumor suppressor genes reprogram cellular metabolism to support rapid cell growth and confer competitive advantages to transformed cells, that is, the Warburg effect.^17, 18^ Thus, we performed an aerobic glycolysis assay to evaluate metabolic changes in KGN cells deficient for *HBP1, TP53*, *WISP1*, *PTEN* or *CTNNA2.* We observed that the extracellular acidification rate (ECAR) significantly increased in KGN cells deficient for *TP53* or *PTEN* (Figures 2c and d, respectively). However, we observed that deficiencies in *HBP1, CTNNA2 or WISP1* did not alter or decrease the ECAR in targeted KGN cells (Figures 2c and d). These data suggest that deficiencies in *HBP1, CTNNA2* and *WISP1* might have important roles during mitochondrial adaptation in KGN cells. Among these genes, the function of *Hbp1*, which is expressed in developing ovaries, remains unknown.^19^ Thus, we focused on *Hbp1* in further functional analyses to verify the results of the screening analyses, and address whether *Hbp1* is associated with mitochondrial regulation and female reproduction *in vivo*.

### *Hbp1* knockout or overexpressing transgenic mice demonstrate significantly altered mitochondrial biogenesis in GCs *in vivo*

To determine the functional consequence of Hbp1-deficiency *in vivo*, we generated conditional knockout mice (Supplementary Figure 3a). The *Hbp1-flox* mice were subsequently crossed with *EIIa-Cre*^20^ to generate *Hbp1* knockout mice (Supplementary Figure 3b).

**Figure 3:**
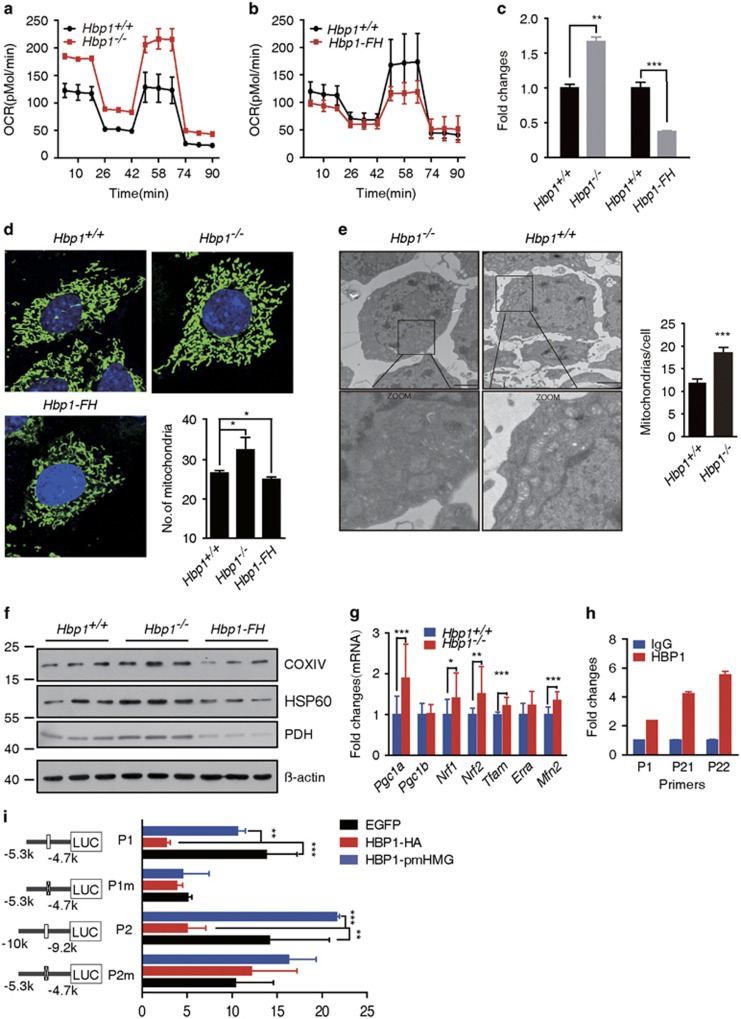
Impacts of *Hbp1* loss- or gain-of-function on mitochondrial respiration. (**a**) Genetic ablation of *Hbp1* increased OCR levels in ovarian GCs. The cells were sequentially treated with oligomycin (1 μM), *p*-trifluoromethoxy carbonyl cyanide phenyl hydrazone (FCCP, 0.5 μM), antimycin A and rotenone (rote, 1 μM), and subsequently monitored using the Seahorse Bioscience Extracellular Flux Analyzer in real time. The data are presented as the mean values±S.D., *n*=3 per group in the Seahorse experiments from three independent experiments. (**b**) OCR levels decreased in *Hbp1*-*FH* GC, when monitored in real time using the Seahorse XF^e^24 as described above. All data are presented as the mean values±S.D., *n*=3 per group in the Seahorse experiments from three independent experiments. (**c**) mtDNA copy number increased in *Hbp1*^−/−^ primary GCs, although decreased in *Hbp1*-FH mice. Genomic DNA (nuclear and mitochondrial) was isolated from ovaries with PMSG for 24 h. Q-PCR was performed to evaluate gene copy of CO1 (mitochondrial) and ß-globin (nuclear), and then the relative mtDNA copy number was shown by mtDNA/nuclear DNA. The data are presented as the mean values±S.D. (*n*=3), **P<0.01; ***P<0.001. (**d**) Mitochondrial morphology in the GCs was indicated using MitoTracker Geeen FM (Invitrogen, Waltham, MA, USA) and the cell nuclei were stained with DAPI after culture for 24 h *in vitro*. Mitochondrial numbers were averaged from >70 GCs per genotype. All data are presented as the mean values±S.D. (*n*=4), *P<0.05. (**e**) Ultrastructural features assessed through transmission electron microscopy in *Hbp1*^*+/+*^ and *Hbp1*^−/−^ ovarian GCs. The boxed areas represent expanded regions (magnified) from panels of the selected region. Right panel showed statistical analysis from three independent experiments. Mitochondrial numbers were quantified in 10–15 cells from random fields. Each column represents mean values±S.D., ***P<0.001. Scale bar, 20 nm. (**f**) Western blot analysis of cytochrome *c* oxidase COXIV, pyruvate dehydrogenase (PDH) and heat-shock protein 60 (HBP60) in *Hbp1*^*+/+*^, *Hbp1*^−/−^and *Hbp1-FH* primary GCs (*n*=3). (**g**) Q-PCR results of genes related to mitochondrial biogenesis and function in *Hbp1*^−/−^ ovaries compared with *Hbp1*^*+/+*^ (wild-type) ovaries at 2-month-old mice. The data are represented as mean values±S.D., *n*=14 for *Hbp1*^−/−^ and *Hbp1*^*+/+*^ ovaries. *P<0.05, **P<0.01 and ***P<0.001. (**h**) Chromatin immunoprecipitation analysis showed Hbp1 binding to the *Tfam* promoter locus in the ovary. P1 is the primer pair for the first Hbp1 affinity sites, and P21 and P22 is the two pair primers for the second Hbp1 affinity site. Each column represents the mean values±S.D. (**i**) Luciferase assay results of Hbp1 modulation on *Tfam* promoter transcriptional repression in KGN cells. The indicated *Tfam* promoter-luciferase constructs were co-transfected with the wild-type *Hbp1* (HBP1-HA) or mutant *Hbp1* (HBP1-pmHMG) cDNA. Each column represents the mean values±S.D., and the results are confirmed by three independent experiments. **P<0.01 and ***P<0.001. P1, first Hbp1 affinity site; P1m, deletion of P1 site; P2, distant Hbp1 affinity site; P2m, deletion of P2 site

Mitochondrial respiration was significantly altered in *Hbp1*-deficient cells, exhibiting a 29% increase in the OCR at baseline and a 59% maximal respiration rate increase (Figure 3a; Supplementary Figure 4a), similar to KGN cells (Figures 2a and b). Together, these results demonstrate that *Hbp1* expression correlates with the changes in mitochondrial respiration.

To address the effects of *Hbp1* gain-of-function on mitochondrial respiration, we generated *Hbp1* transgenic mice using bacterial artificial chromosomes (BACs) harboring a FLAG-HA tag, referred to as *Hbp1-FH* transgenic mice (Supplementary Figure 3c). The basal OCR, maximal respiration rate and ATP capacity were all inhibited (Figure 3b; Supplementary Figure 4b) in GCs obtained from *Hbp1-FH* transgenic mice, further suggesting that Hbp1 dose is a key determinant in mitochondrial respiration.

Then, we examined whether Hbp1 regulates mitochondrial biogenesis through mitochondrial genomic or structural changes. We measured the mitochondrial (mt) DNA content and total mtDNA copy number relative to the diploid chromosomal DNA content using a Q-PCR-based procedure.^21^ We observed that the ratio of mtDNA to nuclear DNA content was notably increased, an ~1.6-fold increase in *Hbp1* knockout cells compared with age-matched wild-type cells (Figure 3c). In contrast, Hbp1 overexpression in *Hbp1-FH* transgenic mice resulted in an ~2.6-fold decrease in mtDNA content (Figure 3c). The effects of both loss- and gain-of-function analyses suggest a consistent role for Hbp1 in altering mtDNA density in GCs. Next, we examined the amount and subcellular localization of mitochondria in GCs using MitoTracker Green, which labels mitochondria. Consistent with mtDNA changes, the amount or distribution of mitochondria in *Hbp1*^−/−^ GCs markedly increased compared with that observed in wild-type or *Hbp1-*overexpressing cells (Figure 3d). We observed that mitochondria were filamentous with a tubular or thread-like appearance and were often interconnected to form a network either in *Hbp1*^−/−^ or *Hbp1*-FH GCs, indicating that disruption of *Hbp1* does not result in mitochondrial fragmentation. We confirmed this result using transmission electron microscopy (TEM; Figure 3e). Furthermore, to gain additional evidence for loss of *Hbp1*-induced generation of mitochondria, the expression level of several proteins normally enriched in mitochondria was investigated in primary GCs. We found that cytochrome *c* oxidase subunit IV (COXIV), pyruvate dehydrogenase (PDH) and heat-shock protein 60 (HSP60) levels increased in *Hbp1*^−/−^ GCs, but were inhibited in *Hbp1-*overexpressing cells (Figure 3f), in concert with the role of Hbp1 for mitochondrial biogenesis.

Moreover, we measured the mRNA levels of the genes implicated in mitochondrial biogenesis in the ovaries, including *Pgc1α*, *Pgc1β*, *Nrf1*, *Nrf2*, *Tfam*, estrogen-related receptor a (*Errα*) and mitofusin (*Mfn2*), at 2-month-old ovaries. *Pgc1α*, *Mfam* and *Tfam* mRNA levels were significantly increased in *Hbp1*^−/−^ mice (Figure 3g), suggesting an altered mitochondrial function. Among these genes, we observed that *Tfam* contained high-affinity Hbp1-binding sites (P1: TTCATTCA and P2: GGGTAG(T)GG) at −9699 and −5098 bp from the transcriptional start, which are the well-characterized Hbp1 response elements.^22, 23^ The results of the chromatin immunoprecipitation (ChIP) analysis revealed the increased enrichment of Hbp1 binding to both sites in the *Tfam* promoter region in the ovaries of *Hbp1-FH* mice (Figure 3h). Luciferase reporter assays showed that either Hbp1-binding site (P1m or P2m) or DNA-binding-defective (HBP1-pmHMG)^24^ mutants abolished Hbp1-mediated regulation (Figure 3i), suggesting that Hbp1 directly regulates *Tfam* transcriptional activity through binding at both sites.

### Genetic ablation of *Hbp1* led to decreased GC apoptosis and increased oocyte production *in vivo*

**Figure 4:**
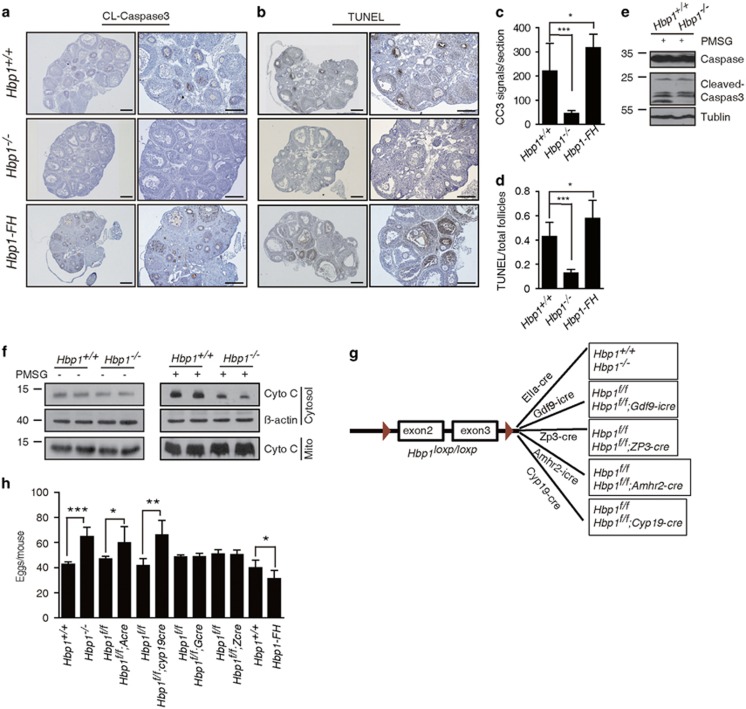
Genetic ablation or transgenic activation of *Hbp1* modulate GC apoptosis and oocyte production during follicle development. (**a**) Immunohistochemistry for cleaved caspase 3 (CC3) in the ovaries of *Hbp1*^*+/+*^, *Hbp1* ^−/−^ and *Hbp1-FH* P21 mice after PMSG treatment for 24 h. The right panel is the enlarged images of the left panel. Scale bar, 100 μm. (**b**) The TUNEL assay for cell death and follicle atresia in the ovaries of *Hbp1*^*+/+*^, *Hbp1*^−/−^ and *Hbp1-FH* P21 mice following PMSG treatment for 24 h. The right panel is the enlarged images of the left. Scale bar, 100 μm. (**c**) Positive signals of CC3 in the ovaries of *Hbp1*^*+/+*^, *Hbp1*^−/−^ and *Hbp1-FH* mice. Ovaries from mice treated with PMSG for 24 h were embedded in paraffin, and sections of 6 μm in thickness were prepared and processed for immunohistochemical analysis, with standard procedure in method. The positive signals in each section were observed under microscope. Each column represents the mean values±S.D. (*n*=6), *P<0.05; ***P<0.001. (**d**) Percentage of follicles exhibited TUNEL-positive signals for each section in ovaries from P21 *Hbp1*^*+/+*^, *Hbp1*^−/−^ and *Hbp1-FH* mice following PMSG treatment for 24 h. Each column represents the mean values±S.D. (*n*=6). *P<0.05; ***P<0.001. (**e**) Western blot assay for pro-caspase 3 and CC3 expression in the ovaries before (left) and after PMSG (right) treatment for 24 h. (**f**) Cytochrome *c* was released in cytosol in response to PMSG treatment for 24 h in *Hbp1*^*+/+*^ and *Hbp1*^−/−^ ovaries. (**g**) Schematic illustration of mouse crosses to generate mouse models with *Hbp1*-specific deficiencies in oocytes or GCs. (**h**) Superovulation assay of mice with different phenotypes to characterize oocyte maturation and determine the numbers of oocytes. Each column represents the mean values±S.D. (*n*=6), *P<0.05; **P<0.01; ***P<0.001

Given the importance of mitochondrial function in apoptotic signaling, we compared the apoptosis levels in the ovaries of 3-week-old wild-type and *Hbp1*^−/−^ mice treated with PMSG for 24 h. We performed terminal deoxynucleotide transferase-mediated deoxyuridine triphosphate nick-end labeling (TUNEL) and immunohistochemistry (IHC) analysis of cleaved caspase 3 (CC3). The results of both CC3-IHC and TUNEL analyses revealed vigorous granulosa cell (GC) apoptosis at various stages of development in the ovaries of wild-type mice (Figures 4a–d), corresponding to the rapid loss of follicles in early stages. In contrast, the numbers of CC3-positive GCs and apoptotic follicles were markedly decreased in the ovaries of PMSG-primed *Hbp1*^−/−^ mice (Figures 4c and d). Western blot analysis revealed that the level of CC3 was significantly attenuated in the ovaries of *Hbp1*^−/−^ mice, whereas the level of pro-caspase 3 remained similar in the ovaries of mutant compared with wild-type mice (Figure 4e). Conversely, we observed an increase in apoptotic cells in the ovaries of *Hbp1*-*FH* transgenic mice (Figures 4a–d). Furthermore, we found that the cytochrome c releases in cytosol were much lower in the *Hbp1*^−/−^ ovaries compared with that in wild-type ovaries in response to PMSG treatment (Figure 4f), suggesting that improved mitochondrial function prevents a key initiating step in the apoptotic process.

We extended the apoptotic analysis to 2-month-old adult mice through immunofluorescence using antibodies against CC3 (Supplementary Figure 5a), cleaved caspase 8 (Supplementary Figure 5b) and poly (ADP-ribose) polymerase (PARP; Supplementary Figure 5c). We observed a marked decrease in the apoptotic cells in secondary and pre-antral follicles in *Hbp1*^−/−^ mice compared with wild-type mice. These results together suggest that the inhibition of *Hbp1* reduces apoptosis in GCs.

To assess whether decreased GC apoptosis contributes to oocyte production, we induced superovulation in indicated genotype mice at P21 (Figures 4g and h). *Hbp1*^−/−^ mice produced more oocytes compared with wild-type littermates (Figure 4h). In contrast, *Hbp1*-*FH* transgenic mice exhibited reduced oocyte maturation (Figure 4h). These results suggest that the gene dosage of *Hbp1* impairs apoptotic resistance in GCs and therefore suppresses oocyte production.

To confirm that the augmented oocyte production reflects the GC-specific disruption of *Hbp1* rather than a systematic effect of the *Hbp1* knockout, we generated *Cyp19-Cre* transgenic mice, exhibiting limited *Cre* expression in GCs and luteal cells.^25^ The *Hbp1*^*f/f*^ mice were bred with the *Cyp19-Cre* line to generate *Hbp1*^*f/f*^; *Cyp19-Cre* mice (Figure 4g; Supplementary Figure 3d). The *Hbp1*^*f/f*^; *Cyp19-Cre* mice exhibited increased ovulation compared with control mice (Figure 4h). Furthermore, the *Hbp1*^*fl/fl*^ mice were crossed with *Gdf-iCre*,^26^ *Zp3-Cre* (*Zcre*)^27^ and *Amhr2-Cre* (*Acre*)^28^ mice to generate the primordial oocyte-, growing oocyte- and granulosa-specific deletion of *Hbp1* in mice, referred to as *Hbp1*^*f/f*^; *Gdf9-iCre* (*Gcre*), *Hbp1*^*f/f*^; *Zcre* and *Hbp1*^*f/f*^; *Acre* mice, respectively (Figures 4g and h). Consistently, *Hbp1*^*f/f*^; *Acre* mice exhibited increased ovulation compared with control mice (Figure 4f). However, the genetic ablation of *Hbp1* in the oocytes of *Gcre* and *Zcre* mice showed less effect on oocyte production compared with control mice (Figures 4g and h). Together, these data demonstrated that the genetic ablation of *Hbp1* in GCs is sufficient to increase oocyte production, consistent with reduced apoptotic signals in GCs and increased mitochondrial function.

### Inhibition of *Hbp1* attenuates granulosa-induced follicle atresia

**Figure 5:**
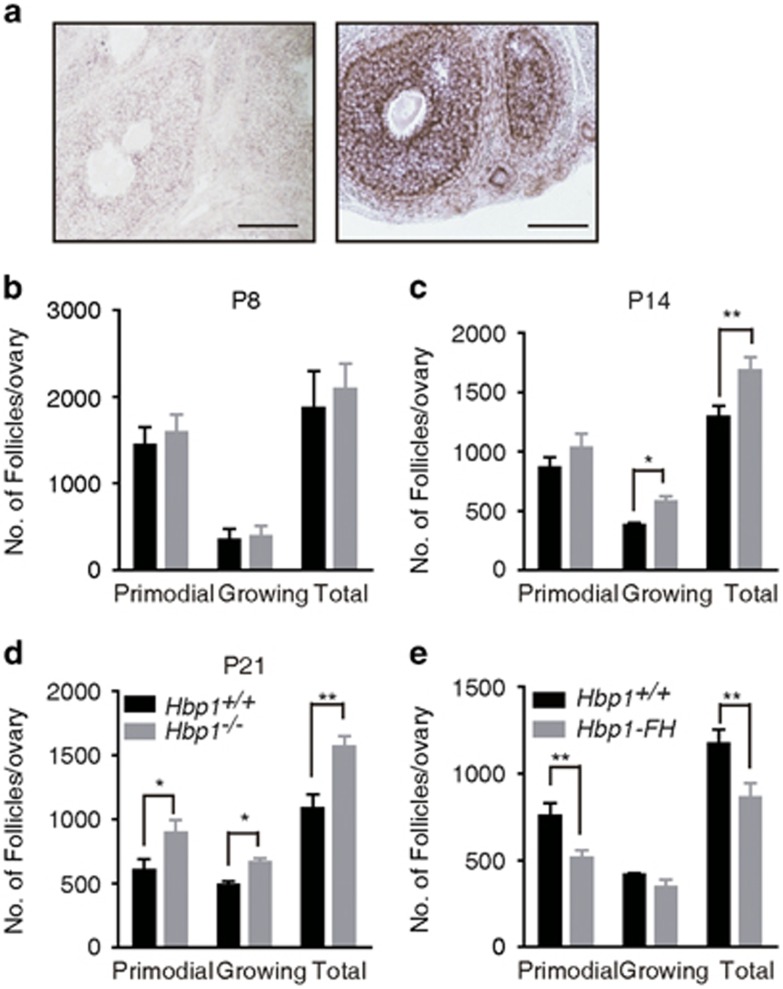
Numbers of ovarian follicles at different developmental stages. (**a**) *In situ* hybridization of an ovarian section from a 21-day-old PMSG-primed mouse, using digoxin-labeled *Hbp1* sense and antisense riboprobes. All follicles in a random region showed similar patterns. The results are confirmed by three independent experiments. Scale bar, 100 μm. (**b**) Ovarian follicle numbers in the ovaries of *Hbp1*^−/−^and *Hbp1*^*+/+*^mice at P8. The data are presented as the mean values±S.E.M. (*n*=6). (**c**) Quantification of the follicle numbers and the follicle development in the ovaries of *Hbp1*^−/−^and *Hbp1*^*+/+*^ littermates at P14. The data are presented as the mean values±S.E.M. (*n*=6). *P<0.05; **P<0.01. (**d**) Ovarian morphology and numbers of follicles in wild-type and *Hbp1*^−/−^ mice at P21. All the data are presented as the mean values±S.E.M. (*n*=6). *P <0.05; **P <0.01. Scale bar, 100 μm. (**e**) Follicle development and numbers of follicles in wild-type and *Hbp1-FH* mice at P21. All the data are presented as the mean values±S.E.M. (*n*=6). **P <0.01. Scale bar, 100 μm

*Hbp1* is intensively in the GCs of primary, secondary and pre-antral follicles (Figure 5a). We next examined the physiological effects through an analysis of follicle dynamics in serial sections of *Hbp1*^*+/+*^ and *Hbp1*^−/−^ ovaries at P8, P14 and P21. The numbers of primordial follicles in the ovaries of wild-type and *Hbp1*^−/−^ mice were similar at either P8 or P14 (Figures 5b and c; Supplementary Figures 6a and b). However, the growing follicles were significantly increased in the ovaries of *Hbp1*-deficient mice at P14 (Figure 5c; Supplementary Figure 6b). Both primordial and growing follicles were significantly increased in the ovaries of *Hbp1*^−/−^ mice at P21 (Figure 5d; Supplementary Figure 6c). Conversely, the follicle number was reduced at P21 in the ovaries of *Hbp1*-*FH* transgenic mice (Figure 5e; Supplementary Figure 6d). These results suggest that Hbp1 represses follicle growth in a gene dosage-dependent manner.

To distinguish the contribution of oocytes and GCs to the increased follicle growth in the absence of *Hbp1*, follicle numbers were examined in three types of *Hbp1* conditional gene-targeting mutants as described above. The numbers of growing follicles in GC-specific *Hbp1*^*f/f*^; *Acre* mice significantly increased at P21 compared with *Hbp1*^*f/f*^ mice, similar to *Hbp1*^−/−^ mice (Supplementary Figures 6e and f). However, no significant differences were noted in the follicle numbers in primordial oocyte-specific *Hbp1*^*f/f*^; *Gcre* or growing oocyte-specific *Hbp1*^*f/f*^; *Zcre* mice compared with *Hbp1*^*f/f*^ mice (Supplementary Figures 6g–j). These results suggest that *Hbp1* regulation in GCs is critical for follicle development.

To determine whether increased follicle growth in *Hbp1*^−/−^ mice reflected increased follicle development, we examined the proliferation rate of GCs in developing follicles after staining cells with DAPI and mitosis-specific phospho-histone H3 (PHH3) in adult mice. We observed no significant difference in the GC proliferation rates between *Hbp1*^−/−^ and wild-type mice (Supplementary Figure 7a). Furthermore, we evaluated the proliferation of primary GCs from wild-type and knockout mice by BrdU incorporation, and found that loss of *Hbp1* did not affect the proliferation in primary culture system (Supplementary Figure 7b). We observed no significant differences in the expression of these genes in the ovaries of wild-type and *Hbp1*^−/−^ mice (Supplementary Figure 7c). These results together suggest that GC proliferation is slightly or less affected after the genetic ablation of *Hbp1*.

### Genetic ablation of *Hbp1* increased ovarian reserve but affects fertility

**Figure 6:**
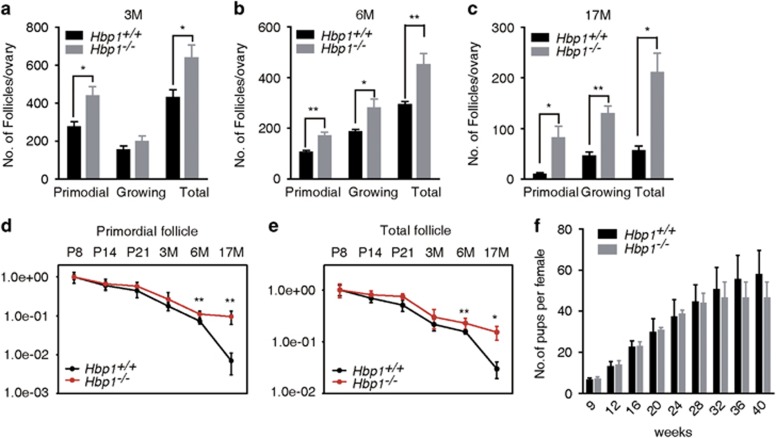
Follicle reserve retention in the ovaries of aging mice. (**a**, **b**) Follicle pool and growing follicles were analyzed at 3 and 6 months old. *n*=5 per phenotype, and the data are presented as the mean values±S.E.M. *P<0.05; **P<0.01. (**c**) Follicle reserve and growing follicles in the ovaries of 17-month-old female mice. Each column presents the mean values±S.E.M. (*n*=6), *P<0.05. **P<0.01. (**d**) Depletion rate of primordial follicles in ovaries from P8 to 17-month-old *Hbp1*^−/−^ and wild-type ovaries. The data are presented as the mean values±S.D. (*n*=6), **P<0.01. (**e**) Depletion rate of total follicles from P8 to 17-month-old *Hbp1*^−/−^ and wild-type ovaries. The data are represented as mean values±S.D. (*n*=6), *P<0.05 and **P<0.01. (**f**) Plot of the cumulative number of pups delivered at parturition for wild-type *Hbp1*^*+/+*^ (*n*=6) and *Hbp1*^−/−^ females (*n*=8). The data are ++-presented as mean values±S.D.

The reduced GC apoptosis and increased follicle growth in *Hbp1*^−/−^ mice at puberty prompted an examination of follicular development in *Hbp1*^−/−^ mice at more mature ages. As expected, the numbers of follicles at various stages in 3- and 6-month-old *Hbp1*^−/−^ mice were significantly increased compared with wild-type controls (Figures 6a and b). We extended this analysis to 17-month-old mice (Figure 6c), observing larger differences in the follicle numbers at various stages between wild-type and *Hbp1*^−/−^ mice with increased aging. These results suggest a cumulative effect of Hbp1 during follicle development. A fixed follicle reserve is progressively depleted during the reproductive lifespan.^29^ In the present study, we observed a significant decrease in the depletion rate of primordial follicles and total follicles at 17 months old in *Hbp1*^−/−^ compared with at 17 months old in the ovaries of wild-type mice (Figures 6d and e). Moreover, increased follicle growth was observed in the ovaries of *Hbp1*^−/−^ mice compared with wild-type mice (Supplementary Table S2), suggesting that the inhibition of Hbp1 attenuates granulosa-induced follicle atresia, thereby increasing follicle growth and alleviating primordial follicle depletion. However, *Hbp1*^−/−^ mice became sterile at 7 months old by unidentified factors (Figure 6f).

To clarify whether the infertility is due to the abnormality in oocyte function or other systematic changes, we did following experiments. First, ovaries from 8-month-old *Hbp1*^−/−^ mice were transplanted into ovariectomized wild-type recipients. The recipient wild-type mice became pregnant and produced pups. In contrast, ovaries from young wild-type mice were transplanted into ovariectomized 8-month-old *Hbp1*^−/−^ mice, and the recipient *Hbp1*^−/−^ mice failed to get pregnant (Supplementary Figure 8a). These results suggest that follicles retained in 8-month-old *Hbp1*^−/−^ mice are healthy. We observed that FSH and LH maintained normal ranges in *Hbp1*^−/−^ females (Supplementary Figures 8b–d) in aging mutant mice. These results suggest that the *Hbp1* deficiency did not alter gonadotropin hormone secretion, but rather decreased follicle depletion in mutant mice.

### Global effects of transcriptional regulation by *Hbp1*

To determine the initial changes in global gene expression induced through Hbp1, a comparative microarray was conducted at P8. We observed that only two (*Sntg2*, 6530401N04Rik) of 1 9051 probes (Roche NimbleGen, Madison, WI, USA) showed markedly changed (>2-fold) between wild-type and *Hbp1*^−/−^ mice, which is consistent with the observation of no abnormal phenotype before P8 in the ovaries of mutant mice.

**Figure 7:**
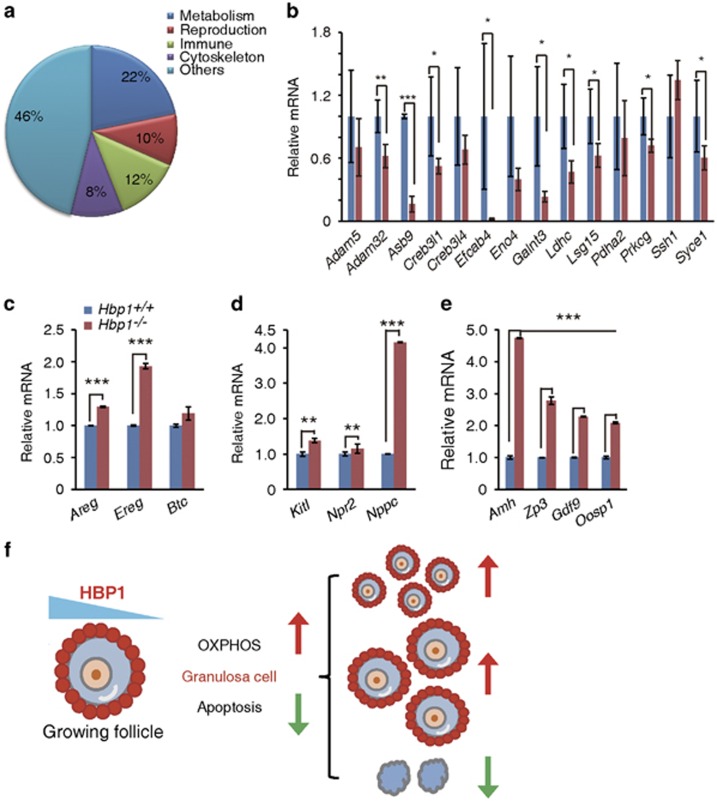
Microarray results of global transcriptional changes and quantitative PCR analysis of metabolic or primordial follicle dormancy-related genes in *Hbp1*-deficient mice. (**a**) Functional classification of the microarray data for the most significantly changed genes in the ovaries of *Hbp1*^−/−^ *versus* wild-type mice of P21. The percentage of genes sharing common biological processes is shown. (**b**) Q-PCR confirmation of metabolic genes showing significant changes in microarray analysis in ovary. Relative mRNA values are normalized to *Rpl19*, and the data are presented as the mean values±S.D. of three independent experiments. (**c–e**) Q-PCR results of EGF ligand family genes (*Areg, Ereg* and *Btc*) that are essential for increased follicle survival, oocyte and primordial follicle dormancy marker genes (*Amh,Zp3,Gdf9* and *Oosp1*), and the genes (*Kitl*, *Npr2* and *Nppc*) essential for communication between oocytes and GCs in the ovaries of 2-month-old mice. Relative mRNA values are normalized to *Rpl19*, and the data are presented as the mean values±S.D. of three independent experiments. (**f**) Model of dose-dependent HBP1 determines the mitochondrial function and therefore contributes to follicle survival and increased ovarian reserve

Next, we compared the global gene expression levels in the ovaries of PMSG-primed wild-type and *Hbp1*^−/−^ mice at P21. We observed that *Hbp1*-deficiency caused a twofold or higher change in the expression of 693 genes (107 upregulated and 585 downregulated). The gene ontology-based classification of these genes revealed that a number of metabolism-related, immune response and reproduction-related genes were regulated (Figure 7a). Notably, most metabolism-related genes were downregulated in the ovaries of *Hbp1*^−/−^ mice, confirmed through Q-PCR (Figure 7b). These results provided valuable genomic information for the relationship between metabolism and early ovarian development, and consequences of ovarian reserve.

We further examined the transcriptional expression in 2-month-old mice. Consistent with improved ovarian reserve in the ovaries of *Hbp1*^−/−^ mice, we observed a marked increase in the transcription of the genes in the EGFR ligand family, including *Areg*, *Ereg* and *Btc*; the folliculogenetic genes, *Amh*, *Zp3*, *Gdf9* and *Oosp1*; the genes responsible for the intracellular signaling between oocytes and GCs, *Kitl* and *Nppc* (Figures 7c–e); and genes associated with mitochondrial function (Figure 3e). These results are in consistent with increased reserve in the ovaries of *Hbp1*^−/−^ mice.

## Discussion

In the present study, we piloted an OCR-based mitochondrial functional screening to evaluate mitochondrial biogenesis in CRISPR/Cas9-edited KGN cells. The results revealed that *Hbp1* inhibition profoundly impacts mitochondrial function and ovarian reserve. These results suggest that improving the bioenergetics of the GCs could improve ovary reserve, and *Hbp1* is an important target in this process.

The candidate genes identified here represent the tip of the iceberg with respect to the regulation of mitochondrial function during folliculogenesis. *TP53* has been reported to regulate energy metabolism in mitochondria by targeting the cytochrome *c* oxidase 2.^30^ Lack of *TP53* led to lower oxygen consumption and increased glycolysis.^30^ However, we unexpectedly found that depletion of *TP53* in the KGN cells displays an obvious increase in mitochondrial respiration and glycolysis, indicating that these cell lines have a remarkably different metabolic behavior. In the present study, we focused on the function of *Hbp1*. Previous studies have indicated that Hbp1 regulates proliferation and senescence, and inhibits Wnt signaling.^31, 32^ Recent evidence suggests that the *Hbp1* gene is frequently mutated in breast cancer^33^ and leukemia.^34^ We provided the first evidence that the genetic ablation of *Hbp1* activated mitochondrial biogenesis without altered glycolysis in contrast to PTEN and TP53. Notably, as the oocyte matures, the need for more energy requires a shift from glycolysis to OXPHOS.^35^ Thus, it is tempting to hypothesize that the effect on mitochondria and glycolysis through *Hbp1* might be responsible for the major biological consequences on ovarian development and reserve, although it remains unclear in the study what causes its infertility in *Hbp1*^−/−^ female.

Given the important role of mitochondria in apoptosis, we examined the effect of mitochondrial function through establishing a genomic and functional screening system in the KGN cells and conducted *in vivo* genetic studies, using mouse models. The results are partially supported through published observations in different systems, demonstrating that the modulation of mitochondrial biogenesis is associated with the sensitivity of cell death,^36^ and stimulating production of new mitochondria could suppress the aging process in the brain.^37^ In this manner, we observed that *Hbp1* regulates *Tfam* mRNA expression. TFAM not only regulates mtDNA copy and transcription levels, but also stabilizes the mitochondrial genome through substituting histone function.^38, 39^ These functions contribute to the favorable cell survival in response to environmental stress. We therefore reasoned that increased mitochondrial biogenesis in *Hbp1*^−/−^ mice at least in part contributes to GC survival, resulting in the decreased GC apoptosis and refined conditions favoring the survival of developing follicles (Figure 7f). We acknowledge that these phenotypes might be due to pleiotropic effects of Hbp1.

## Materials and Methods

### Construct design for CRISPR in human cells

The plasmids for expression of *Streptococcus pyogenes* Cas9 and sgRNA were described previously.^40^ In brief, the Cas9 expression plasmid pST1374-Cas9-N-NLS-Flag-linker (Addgene 44758, Cambridge, MA, USA) has been optimized for nuclear import by two NLS and contains a CMV promoter for expression in human cells. Furthermore, this plasmid also contains a blasticidin selection cassette for obtaining transfected cells expressing Cas9. The sgRNA plasmid (pGL3-U6-sgRNA-PGK-Puro) contains a puromycin selection cassette and a high-fidelity restriction enzyme BsaI for linearization. This plasmid allows two complementary single-strands DNA annealing to obtain the sgRNA, containing linkers for ligating to the expression vector. The sgRNA design is as the following rules, the forward strand is 5′-ccgg (N)_18–20_-3′ and the reverse strand is 5′-aaac(N)_18–20_-3′.

### Cell culture, DNA transfection and deletion efficiency assay

The KGN cells were cultured in Dulbecco's modified Eagle Medium/F12 (DMEM/F12) medium containing 10% fetal bovine serum (FBS) in a six-well plate. For transient transfection experiments, cells were transfected with 2 μg Cas9 expression plasmid and 1 μg two sgRNA expression plasmid (0.5 μg each) for DNA fragment deletion or double digestion by lipofectmine 3000 (Invitrogen). After 36–48 h, the selection antibiotics blasticidin (5 μg/ml) and puromycin (1 μg/ml) were added to cells to select cells expressing Cas9 and sgRNA, and maintained in cells for 48 h. Five days later, the antibiotics were added to cells again and maintained for 48 h. After cell clones formed, the cells were taken for detecting deletion efficiency and functional assay. Medium was changed every 2–3 days. To detect deletion deficiency, primers spanning the two sgRNAs region, including the sgRNA locus were selected. The criterion of primer design was exhibited in Supplementary Figure S1a.

### Mouse strains

The *Hbp1* conditional knockout mice were generated through the deletion of the exons 2 and 3 through the insertion of loxp into the intron (Supplementary Figure S3A). The positive ES cells were screened using PCR and confirmed through Southern blotting, generating *Hbp1*^*loxp/loxp*^ mice. Subsequently, *Hbp1*^*loxp/loxp*^ mice were crossed with transgenic mice carrying *EIIA* promoter-mediated Cre recombinase (*EIIA*-cre) to generate knockout mice (*Hbp1*^−/−^) and crossed with Gdf9 promoter-mediated Cre recombinase (*Gcre*) to generate oocyte mutant mice (*Hbp1*^*f/f*^*; Gcre*), both of which had a C57BL/6J background. The knock-in *Amhr2* promoter-mediated Cre recombinase mice (*Acre*) and transgenic mice *Cyp19*-cre were crossed with the *Hbp1*^*loxp/loxp*^ strain to generate mutant female mice, lacking *Hbp1* in GCs (*Hbp1*^*f/f*^*; Acre* and *Hbp1*^*f/f*^*; Cyp19-cre*). The control mice that did not carry Cre recombinase were referred to as *Hbp1*^*f/f*^. BAC transgenic mice of Hbp1 carrying a FLAG-HA tag for immunoassays, referred to as *Hbp1-FH* (Figure D3B), were generated through pronuclear injection. All mice were housed in specific pathogen-free experimental conditions with free access to water and food. The light was on between 08:00 and 20:00 h. The animal experimental protocols were approved and examined through the regional ethical committee of Model Animal Research Center, Nanjing University.

### GC isolation and culture

Immature female mice at 23–28 days were injected intraperitoneally (i.p.) with 5 IU of PMSG to stimulate follicular growth and GC division. After 24 h, undifferentiated GCs were released from antral follicles after puncturing with a 26.5-Gauge needle. The cells were cultured in DMEM/F12 medium containing 5% FBS, 100 μg/ml of penicillin and 100 μg/ml of streptomycin in 35-mm culture dishes.^41^ After overnight culture, the GCs were seeded onto 24-well plates for XF24 in 250 μl of growth medium and incubated with 5% CO_2_ overnight.

### OXPHOS assay

Cellular OXPHOS was monitored using a Seahorse Bioscience Extracellular Flux Analyzer (XF24e, Seahorse Bioscience) in real time by measuring the OCR, indicating respiration as previously described. In brief, cell numbers were quantified with Moxi Z cell counter (ORFLO, USA), then 50 000 cells were seeded onto 24-well plates for XF24 in 250 μl of growth medium and incubated with 5% CO_2_ for ~6–7 h when the cell attached to wells. Before obtaining the measurements, the cells were washed three times with Seahorse Assay Medium and immersed in 525 μl of assay medium followed by incubation at 37 °C for 1 h in the absence of CO_2_. The OCR was subsequently measured in a typical 8-min cycle of mix (2–4 min), dwell (2 min) and measure (2–4 min) according to the manufacturer's instructions. The final results were normalized according to cells number. The basal levels of OCR were recorded followed by the OCR levels after the injection of individual compounds, inhibiting respiratory mitochondrial electron transport chain complexes. The first inhibitor was oligomycin, which inhibits mitochondrial ATP synthesis. The second inhibitor was *p*-trifluoromethoxy carbonyl cyanide phenyl hydrazone, which uncouples OXPHOS and can be used to calculate the highest respiratory capacity of the cells. The third inhibitor was a combination of rotenone, a complex I inhibitor and antimycin A, a complex III inhibitor, to completely block mitochondrial respiration. All the chemicals were purchased from Seahorse Bioscience.

### Glycolysis assay

Cellular glycolysis was monitored using the Seahorse Bioscience Extracellular Flux Analyzer (XF24e, Seahorse Bioscience) in real time based on measurements of the ECAR, indicating glycolysis as previously described. The assay medium for glycolysis is DMEM-modified XF assay medium containing 0.5 mM sodium pyruvate and 17.5 mM d-glucose. The cells were sequentially treated with glucose (100 mM), oligomycin (oligo, 1 μM) and 2-deoxyglucose (100 mM). The ECAR was subsequently measured in a typical 8- min cycle of mix (2–4 min), dwell (2 min) and measure (2–4 min) according to the manufacturer's instructions. All the chemicals were purchased from Seahorse Bioscience.

### Microscopic analysis of mitochondrial number and structure

The cells were cultured on four-well dishes with glass bottoms and incubated with MitoTracker Green (100 nM) (Invitrogen) at 37 °C for 15 min with 5% CO_2_. Subsequently, the cells were washed with phosphate-buffered saline (PBS) and fixed in 4% formaldehyde for 30 min at 37 °C followed by washing thrice with PBS. Nuclear morphology was analyzed using the fluorescent dye DAPI. The images were obtained with an Olympus Fluoview 1000 confocal microscope (Olympus Corp., Tokyo, Japan). To obtain a volumetric measurement, *z* axis were acquired in 10 panels with 0.6 μm for each step. Collected images were analyzed by the Image J software (NIH, Bethesda, MD, USA). The volumetric object count were obtained by the ‘object count 3D' plugin and the numbers were analyzed by ‘mito morphology' plugin as described previously.^42, 43^

### TEM

Ovary tissues were fixed in 2.5% glutaraldehyde at 4 °C overnight for TEM, then a single dose of tissue was further processed for standard TEM technique with Hitachi 100KV (Hitachi, Tokyo, Japan).

### Relative mtDNA copy number determination

Immature female mice at 23–28 days were i.p. injected with 5 IU of PMSG to stimulate follicular growth and GC division. After 24 h, the ovaries were isolated and placed in homogenizer containing 800 μl of lysis buffer (10 mM Tris–HCl (pH 8.0), 1 mM EDTA and 0.1% SDS), and homogenized using a Dounce homogenizer until no solid pieces were visible. After adding 80 μl of 15 mM proteinase K solution, the lysate was incubated at 55 °C for 2–3 h. The lysate solutions were vigorously vortexed, and the non-soluble fraction was pelleted through centrifugation. Subsequently, total DNA was isolated using the phenol/chloroform/isoamyl alcohol (25:4:1) method as previously described.^44^ The mtDNA gene cytochrome *c* oxidase subunit I (CO1) and nuclear DNA β-globin gene were amplified through Q-PCR (ABI StepOne Fast Real-Time PCR System, Carlsbad, CA, USA). The CO1 primers were 5′-*TGCTAGCCGCAGGCATTAC*-3′ (forward primer) and 5′-*GGGTGCCCAAAGAATCAGAAC*-3′ (reverse primer). The β-globin primers were the same as previously described.^44^

### BrdU incorporation assay

Cells in coating dishes were fixed with 2% paraformaldehyde after incubated with 3 μg/ml BrdU for 2 h. Incorporated BrdU was detected by immunofluorescence using a BrdU antibody (Sigma, St. Louis, MO, USA) according to manufacturer's instructions.

### Chromatin immunoprecipitation and reporter gene assays

ChIP assays were performed as described previously,^45^ and hypotonic buffer (Active Motif; 100505) was used to obtain the nuclear extracts. Anti-HBP1 antibody was generated by synthetic peptides as immunogens (Signalway antibody, Baltimore, MD, USA). Rabbit IgGs from nonimmunized rabbits were used as the negative control. All of the primers were validated for SYBR-based real-time PCR (Supplementary Table S6).

The Hbp1 cDNA and the mutant Hbp1-pmHMG cDNA were cloned into pCGN with HA tag. And the Hbp1 affinity sites among *Tfam* promoter were cloned into PGL3 enhancer vector with luciferase reporter. The luciferase gene reporter assays were performed according to manufacturer's instructions from Promega (Fitchburg, WI, USA). In brief, KGN cells cultured overnight were transfected with these plasmids with lipofectamine 3000 (Invitrogen) as instructions. The transfection efficiency was measured by cotransfecting Renilla luciferase expression vector, and the firefly luciferase activity results were normalized to enzymatic Renilla luciferase activity.

### Histological analysis and quantification of follicles

Ovaries from different ages of mice were fixed with Bouin's fixation solution for 3 h at room temperature followed by dehydration and embedding in paraffin. Longitudinal sections (7-μm thick) were continuously processed and flattened onto glass. To observe the morphology under the microscope, the sections were stained with hematoxylin and eosin. Subsequently, the quantification of ovarian follicles was performed as previously described. In brief, the follicles were specified as primordial follicles with flattened GCs, and the growing follicles were defined as follicles that have several cuboidal GCs surrounding enlarged oocytes. Follicles containing oocytes with clearly visible nuclei were scored throughout the entire ovary in each section every fifth section among the serial sections, as previously reported.^46^

### Gonadotropin-induced ovulation assay

To synchronize follicle growth and induce ovulation, immature 22- to 25-day-old female mice were i.p. injected with 5 IU of PMSG to stimulate follicular development and 5 IU of human chorionic gonadotrophin (hCG) at 48 h after PMSG treatment to induce ovulation. After hCG stimulation for 15–16 h, cumulus–oocyte complexes were collected from the oviducts and incubated with hyaluronidase (1 mg/ml) to separate the oocytes for counting.

### Histochemistry and western blot

For histochemistry, the ovaries were collected and fixed in 4% paraformaldehyde PBS for 3 h at room temperature or at 4 °C overnight. After fixation, the ovaries were dehydrated with alcohol and embedded in paraffin, followed by sectioning (6-μm thickness). Subsequently, the ovary sections were deparaffinized in dimethylbenzene, rehydrated in sequential alcohol concentrations and washed with PBS. The sections were incubated in blocking buffer (PBS containing 0.05% Triton-X100 and 10% goat serum) for 1 h at room temperature followed by incubation with primary antibody overnight at 4 °C. The sections were incubated with secondary antibodies at room temperature for 1 h. CC3, caspase 8 and PARP were detected using antibodies diluted 1:500 (Cell Signal Technology, Danvers, MA, USA). PHH3 (Ser10) was detected using an antibody diluted 1:400 (Signalway Antibody). The samples for IHC were processed according to the manufacturer's instructions.

For western blot, protein extracted from ovary tissues were resolved by SDS-PAGE and transferred to PVDF membrane. The membranes were incubated primary, peroxidase-linked secondary and visualized using ECL Substrate (Millipore, Billerica, MA, USA). The antibodies were caspase 3 (1:1000), CC3 (1:500), COXIV (1:1000), PDH (1:1000), HSP60 (1:1000), cytochrome *c* (1:2000, Abcam, Cambridge, MA, USA) and TUBLIN (1:1000; Cell Signaling Technology).

### Ovary transplantation

The recipients for ovarian orthotopic transplantation were 2-month-old C57BL/6J females. The whole ovaries from donors were transplanted to bursa. After 14 days, the recipients were mated with 2-month-old C57BL/6J males.^47^

### TUNEL assay

Cell apoptosis and follicle atresia in female mice were detected using the ApopTag (Billerica, MA, USA) Plus Peroxidase *In Situ* Apoptosis Detection Kit according to the manufacturer's instructions (Millipore).

### Measurement of serum hormone levels

Adult female mice and control females (*n*=3 per genotype) were housed together in the same cage from 21 days, and whole blood was isolated through puncture of the orbital venous plexus of 6-month-old female mice. Subsequently, the blood was incubated at room temperature for 30 min and centrifuged for 10 min at 4 000 r.p.m. Subsequently, the serum was collected and centrifuged a second time. The serum was stored in −80 °C. The gonadotropin level was determined through immunoassay as previously described.^48^ For ROS measurement in ovary, tissues were homogenized in PBS on ice, then the samples were centrifuged and the supernatant was saved for ROS analysis through immunoassay according to manufacturer's instructions.

### RNA isolation and real-time PCR

Total RNA was collected from ovaries of normal control and mutant mice using TRIzol reagent (Invitrogen). Reverse transcription was performed using the PrimeScript RT-PCR Kit. Subsequently, quantitative real-time PCR was performed in triplicate using SYBR Green Master Mix (Takara, Otsu, Shiga, Japan) with Stepone Plus (Applied Biosystems, Carlsbad, CA, USA). The relative mRNA levels were calculated according to the ^ΔΔ^Ct principle, normalized to endogenous housekeeping gene ribosome protein L (*Rpl19*) in the same samples. The primer sequences were available at Supplementary Table S3–S6.

### Mitochondrial isolation

The cells (5 × 10^6^) were collected by centrifugation at 650 *g* for 10 min at 4 °C, then the cells were washed twice with cold PBS and resuspended with five volumes of lysis buffer (20 mM Hepes–KOH, pH 7.5, 10 mM KCl, 1.5 mM MgCl2, 1 mM sodium EDTA, 1 mM sodium EGTA, 1 mM dithiothreitol and 0.1 mM phenylmethylsulfonylfluoride) containing 250 mM sucrose. The cells were homogenized for 20 stokes and the homogenates were centrifuged twice 650 *g* for 10 min at 4 °C. The supernatants were centrifuged at 11 000 *g* for 15 min at 4 °C, then resulting mitochondrial pellet were resuspended with mitochondrial lysis buffer. The supernatants were the cytosolic protein.

### Statistical analysis

A student *t*-test was performed using Graphpad Prism software (Graphpad, Inc., La Jolla, CA, USA) to determine the significances between experimental and normal control groups. A value of *P*<0.05 was considered statistically significant (*P<0.05, **P<0.01 and ***P<0.001).

## Abbreviations

GCs: granulosa cells
Hbp1: HMG-box transcription factor 1
Q-PCR: quantitative RT-PCR
EGFP: enhanced green fluorescent protein
OXPHOS: oxidative phosphorylation
OCR: oxygen consumption rate
ECAR: extracellular acidification rate
Acre: Amhr2-cre
Gcre: Gdf9-icre
Zcre: Zp3-cre
PMSG: pregnant mare's serum gonadotropin
hCG: human chorionic gonadotrophin

## References

[bib1] Matzuk MM, Burns KH, Viveiros MM, Eppig JJ. Intercellular communication in the mammalian ovary: oocytes carry the conversation. Science 2002; 296: 2178–2180. 12077402 10.1126/science.1071965

[bib2] Phillipps HR, Hurst PR. XIAP: a potential determinant of ovarian follicular fate. Reproduction 2012; 144: 165–176. 22653317 10.1530/REP-12-0142

[bib3] Wang X. The expanding role of mitochondria in apoptosis. Genes Dev 2001; 15: 2922–2933. 11711427

[bib4] Reddy P, Liu L, Adhikari D, Jagarlamudi K, Rajareddy S, Shen Y et al. Oocyte-specific deletion of Pten causes premature activation of the primordial follicle pool. Science 2008; 319: 611–613. 18239123 10.1126/science.1152257

[bib5] Adhikari D, Flohr G, Gorre N, Shen Y, Yang H, Lundin E et al. Disruption of Tsc2 in oocytes leads to overactivation of the entire pool of primordial follicles. Mol Huma Reprod 2009; 15: 765–770. 10.1093/molehr/gap092 19843635

[bib6] Zoncu R, Efeyan A, Sabatini DM. mTOR: from growth signal integration to cancer, diabetes and ageing. Nat Rev Mol Cell Biol 2011; 12: 21–35. 21157483 10.1038/nrm3025 PMC3390257

[bib7] Hollander MC, Blumenthal GM, Dennis PA. PTEN loss in the continuum of common cancers, rare syndromes and mouse models. Nat Rev Cancer 2011; 11: 289–301. 21430697 10.1038/nrc3037 PMC6946181

[bib8] Nishi Y, Yanase T, Mu Y, Oba K, Ichino I, Saito M et al. Establishment and characterization of a steroidogenic human granulosa-like tumor cell line, KGN, that expresses functional follicle-stimulating hormone receptor. Endocrinology 2001; 142: 437–445. 11145608 10.1210/endo.142.1.7862

[bib9] Shalem O, Sanjana NE, Hartenian E, Shi X, Scott DA, Mikkelsen TS et al. Genome-scale CRISPR-Cas9 knockout screening in human cells. Science 2014; 343: 84–87. 24336571 10.1126/science.1247005 PMC4089965

[bib10] Rosario R, Araki H, Print CG, Shelling AN. The transcriptional targets of mutant FOXL2 in granulosa cell tumours. PloS One 2012; 7: e46270. 23029457 10.1371/journal.pone.0046270 PMC3460904

[bib11] Nagaraja AK, Middlebrook BS, Rajanahally S, Myers M, Li Q, Matzuk MM et al. Defective gonadotropin-dependent ovarian folliculogenesis and granulosa cell gene expression in inhibin-deficient mice. Endocrinology 2010; 151: 4994–5006. 20739397 10.1210/en.2010-0428 PMC2946151

[bib12] Caelles C, Helmberg A, Karin M. p53-dependent apoptosis in the absence of transcriptional activation of p53-target genes. Nature 1994; 370: 220–223. 8028670 10.1038/370220a0

[bib13] Marchenko ND, Zaika A, Moll UM. Death signal-induced localization of p53 protein to mitochondria. A potential role in apoptotic signaling. J Biol Chem 2000; 275: 16202–16212. 10821866 10.1074/jbc.275.21.16202

[bib14] Vaseva AV, Moll UM. The mitochondrial p53 pathway. Biochim Biophys Acta 2009; 1787: 414–420. 19007744 10.1016/j.bbabio.2008.10.005 PMC2819081

[bib15] Liang H, He S, Yang J, Jia X, Wang P, Chen X et al. PTENalpha, a PTEN isoform translated through alternative initiation, regulates mitochondrial function and energy metabolism. Cell Metab 2014; 19: 836–848. 24768297 10.1016/j.cmet.2014.03.023 PMC4097321

[bib16] Su F, Overholtzer M, Besser D, Levine AJ. WISP-1 attenuates p53-mediated apoptosis in response to DNA damage through activation of the Akt kinase. Genes Dev 2002; 16: 46–57. 11782444 10.1101/gad.942902 PMC155313

[bib17] Levine AJ, Puzio-Kuter AM. The control of the metabolic switch in cancers by oncogenes and tumor suppressor genes. Science 2010; 330: 1340–1344. 21127244 10.1126/science.1193494

[bib18] DeBerardinis RJ, Lum JJ, Hatzivassiliou G, Thompson CB. The biology of cancer: metabolic reprogramming fuels cell growth and proliferation. Cell Metab 2008; 7: 11–20. 18177721 10.1016/j.cmet.2007.10.002

[bib19] Smith JM, Bowles J, Wilson M, Koopman P. HMG box transcription factor gene Hbp1 is expressed in germ cells of the developing mouse testis. Dev Dyn 2004; 230: 366–370. 15162515 10.1002/dvdy.20053

[bib20] Lakso M, Pichel JG, Gorman JR, Sauer B, Okamoto Y, Lee E et al. Efficient *in vivo* manipulation of mouse genomic sequences at the zygote stage. Proc Natl Acad Sci USA 1996; 93: 5860–5865. 8650183 10.1073/pnas.93.12.5860 PMC39152

[bib21] Hughes FM Jr, Gorospe WC. Biochemical identification of apoptosis (programmed cell death) in granulosa cells: evidence for a potential mechanism underlying follicular atresia. Endocrinology 1991; 129: 2415–2422. 1935775 10.1210/endo-129-5-2415

[bib22] Zhuma T, Tyrrell R, Sekkali B, Skavdis G, Saveliev A, Tolaini M et al. Human HMG box transcription factor HBP1: a role in hCD2 LCR function. EMBO J 1999; 18: 6396–6406. 10562551 10.1093/emboj/18.22.6396 PMC1171702

[bib23] Tevosian SG, Shih HH, Mendelson KG, Sheppard KA, Paulson KE, Yee AS. HBP1: a HMG box transcriptional repressor that is targeted by the retinoblastoma family. Genes Dev 1997; 11: 383–396. 9030690 10.1101/gad.11.3.383

[bib24] Paulson KE, Rieger-Christ K, McDevitt MA, Kuperwasser C, Kim J, Unanue VE et al. Alterations of the HBP1 transcriptional repressor are associated with invasive breast cancer. Cancer Res 2007; 67: 6136–6145. 17616670 10.1158/0008-5472.CAN-07-0567

[bib25] Fan HY, Shimada M, Liu Z, Cahill N, Noma N, Wu Y et al. Selective expression of KrasG12D in granulosa cells of the mouse ovary causes defects in follicle development and ovulation. Development 2008; 135: 2127–2137. 18506027 10.1242/dev.020560 PMC3541831

[bib26] Lan ZJ, Xu X, Cooney AJ. Differential oocyte-specific expression of Cre recombinase activity in GDF-9-iCre, Zp3cre, and Msx2Cre transgenic mice. Biol Reprod 2004; 71: 1469–1474. 15215191 10.1095/biolreprod.104.031757

[bib27] Lewandoski M, Wassarman KM, Martin GR. Zp3-cre, a transgenic mouse line for the activation or inactivation of loxP-flanked target genes specifically in the female germ line. Curr Biol 1997; 7: 148–151. 9016703 10.1016/s0960-9822(06)00059-5

[bib28] Jamin SP, Arango NA, Mishina Y, Hanks MC, Behringer RR. Requirement of Bmpr1a for Mullerian duct regression during male sexual development. Nat Genet 2002; 32: 408–410. 12368913 10.1038/ng1003

[bib29] Reddy P, Zheng W, Liu K. Mechanisms maintaining the dormancy and survival of mammalian primordial follicles. Trends Endocrinol Metab 2010; 21: 96–103. 19913438 10.1016/j.tem.2009.10.001

[bib30] Matoba S, Kang JG, Patino WD, Wragg A, Boehm M, Gavrilova O et al. p53 regulates mitochondrial respiration. Science 2006; 312: 1650–1653. 16728594 10.1126/science.1126863

[bib31] Sampson EM, Haque ZK, Ku MC, Tevosian SG, Albanese C, Pestell RG et al. Negative regulation of the Wnt-beta-catenin pathway by the transcriptional repressor HBP1. EMBO J 2001; 20: 4500–4511. 11500377 10.1093/emboj/20.16.4500 PMC125566

[bib32] Zhang X, Kim J, Ruthazer R, McDevitt MA, Wazer DE, Paulson KE et al. The HBP1 transcriptional repressor participates in RAS-induced premature senescence. Mol Cell Biol 2006; 26: 8252–8266. 16966377 10.1128/MCB.00604-06 PMC1636767

[bib33] Yee AS, Paulson EK, McDevitt MA, Rieger-Christ K, Summerhayes I, Berasi SP et al. The HBP1 transcriptional repressor and the p38 MAP kinase: unlikely partners in G1 regulation and tumor suppression. Gene 2004; 336: 1–13. 15225871 10.1016/j.gene.2004.04.004

[bib34] Munoz-Alonso MJ, Acosta JC, Richard C, Delgado MD, Sedivy J, Leon J. p21Cip1 and p27Kip1 induce distinct cell cycle effects and differentiation programs in myeloid leukemia cells. J Biol Chem 2005; 280: 18120–18129. 15746092 10.1074/jbc.M500758200

[bib35] Van Blerkom J. Mitochondria in human oogenesis and preimplantation embryogenesis: engines of metabolism, ionic regulation and developmental competence. Reproduction 2004; 128: 269–280. 15333778 10.1530/rep.1.00240

[bib36] Christie DA, Lemke CD, Elias IM, Chau LA, Kirchhof MG, Li B et al. Stomatin-like protein 2 binds cardiolipin and regulates mitochondrial biogenesis and function. Mol Cell Biol 2011; 31: 3845–3856. 21746876 10.1128/MCB.05393-11 PMC3165718

[bib37] Ames BN, Liu J. Delaying the mitochondrial decay of aging with acetylcarnitine. Ann NY Acad Sci 2004; 1033: 108–116. 15591008 10.1196/annals.1320.010

[bib38] Ekstrand MI, Falkenberg M, Rantanen A, Park CB, Gaspari M, Hultenby K et al. Mitochondrial transcription factor A regulates mtDNA copy number in mammals. Hum Mol Genet 2004; 13: 935–944. 15016765 10.1093/hmg/ddh109

[bib39] Scarpulla RC. Transcriptional paradigms in mammalian mitochondrial biogenesis and function. Physiol Rev 2008; 88: 611–638. 18391175 10.1152/physrev.00025.2007

[bib40] Shen B, Zhang W, Zhang J, Zhou J, Wang J, Chen L et al. Efficient genome modification by CRISPR-Cas9 nickase with minimal off-target effects. Nat Methods 2014; 11: 399–402. 24584192 10.1038/nmeth.2857

[bib41] Fan HY, Liu Z, Shimada M, Sterneck E, Johnson PF, Hedrick SM et al. MAPK3/1 (ERK1/2) in ovarian granulosa cells are essential for female fertility. Science 2009; 324: 938–941. 19443782 10.1126/science.1171396 PMC2847890

[bib42] Garcia-Cao I, Song MS, Hobbs RM, Laurent G, Giorgi C, de Boer VC et al. Systemic elevation of PTEN induces a tumor-suppressive metabolic state. Cell 2012; 149: 49–62. 22401813 10.1016/j.cell.2012.02.030 PMC3319228

[bib43] Dagda RK, Cherra SJ 3rd, Kulich SM, Tandon A, Park D, Chu CT. Loss of PINK1 function promotes mitophagy through effects on oxidative stress and mitochondrial fission. J Biolog Chem 2009; 284: 13843–13855. 10.1074/jbc.M808515200 PMC2679485 19279012

[bib44] Guo W, Jiang L, Bhasin S, Khan SM, Swerdlow RH. DNA extraction procedures meaningfully influence qPCR-based mtDNA copy number determination. Mitochondrion 2009; 9: 261–265. 19324101 10.1016/j.mito.2009.03.003 PMC2798162

[bib45] Shi G, Xing L, Liu Z, Qu Z, Wu X, Dong Z et al. Dual roles of FBXL3 in the mammalian circadian feedback loops are important for period determination and robustness of the clock. Proc Natl Acad Sci USA 2013; 110: 4750–4755. 23471982 10.1073/pnas.1302560110 PMC3606995

[bib46] Johnson J, Canning J, Kaneko T, Pru JK, Tilly JL. Germline stem cells and follicular renewal in the postnatal mammalian ovary. Nature 2004; 428: 145–150. 15014492 10.1038/nature02316

[bib47] Sztein J, Sweet H, Farley J, Mobraaten L. Cryopreservation and orthotopic transplantation of mouse ovaries: new approach in gamete banking. Biol Reprod 1998; 58: 1071–1074. 9546742 10.1095/biolreprod58.4.1071

[bib48] Pelosi E, Omari S, Michel M, Ding J, Amano T, Forabosco A et al. Constitutively active Foxo3 in oocytes preserves ovarian reserve in mice. Nat Commun 2013; 4: 1843. 23673628 10.1038/ncomms2861 PMC4504230

