# Supplementary material for: Focused screening of mitochondrial metabolism reveals a crucial role for a tumor suppressor Hbp1 in ovarian reserve
Source: Cell Death Differ. 2016 May 20;23(10):1602–14. doi: 10.1038/cdd.2016.47 (PMC5041189; doi:10.1038/cdd.2016.47)
Supplement: Supplementary Tables [file cdd201647x1.docx]

**Supplementary Table**

**Table S1 Changes of oxygen consumption rate in loss-of-function KGN cell strains**

OCR (Oxygen Consumption Rate), indicative of OXPHOS, were monitored by using the

Seahorse Bioscience Extracellular Flux Analyzer in real time. Cells were treated sequentially

with oligomycin (Olig, 1 μM), p-trifluoromethoxy carbonyl cyanide phenyl hydrazone

(FCCP, 0.5μM), antimycin A (1μM) and rotenone (Rote, 1μM). Vertical lines indicate time points

of the administration of corresponding inhibitors.

The digit means number of cell strains for analysis. The “+” means increase, and the “-”means decrease.

The n.s means no significance, *p<0.05, **p<0.01, ***p<0.005.

| Genes | Selected Cell strains | Basal level | Maximal respiration | ATP production capacity |
| --- | --- | --- | --- | --- |
| *TP53* | 3 | 3+(**) | 3+(**) | 3+(**) |
| *WT1* | 2 | n.s | n.s | 1-(*) |
| *STK11* | 2 | 1+(*) | n.s | n.s |
| *HBP1* | 4 | 3+(**) | 3+(**) | 3+(**) |
| *BRCA1* | 3 | 2+(*) | n.s | n.s |
| *KRAS* | 2 | n.s | n.s | n.s |
| *CTNNA1* | 2 | n.s | n.s | n.s |
| *CTNNA2* | 3 | 2+(**) | 2+(*) | 2+(*) |
| *PTEN* | 2 | 2+(***) | 2+(***) | 2+(***) |
| *PTCH1* | 3 | 1+(*) | 1+(*) | 1+(*) |
| *EXT1* | 2 | n.s | n.s | n.s |
| *BMPR1A* | 2 | 1+(*) | n.s | n.s |
| *KRAS* | 1 | n.s | n.s | n.s |
| *WISP1* | 3 | 2+(*) | 2+(***) | 2+(***) |
| *WRN* | 3 | 2-(*) | 2-(*) | n.s |
| *TSC1* | 3 | n.s | 1+(*) | 1+(*) |
| *TSC2* | 3 | n.s | 2+(**) | 1+(**) |
| *SPOP* | 2 | 1-(*) | n.s | 1-(*) |
| *CHKA* | 3 | 1-(*) | 2-(*) | 2-(*) |
| *CHKB* | 2 | 1+(*) | 1+(*) | n.s |
| *NBN* | 2 | 2-(***) | 2-(***) | 2-(***) |
| *SMAD3* | 3 | 1+(*) | 1+(*) | 1+(*) |
| *SMAD4* | 3 | 1-(*) | 1-(*) | 1-(*) |
| *GPC3* | 2 | n.s | 1-(*) | n.s |

**Table S2 Numbers of follicles in ovaries of different developmental stages**

|  | P8 | | P14 | | P21 | | 3M | | 6M | | 17M | |
| --- | --- | --- | --- | --- | --- | --- | --- | --- | --- | --- | --- | --- |
|  | *Hbp1^+/+^* | *Hbp1^-/-^* | *Hbp1^+/+^* | *Hbp1^-/-^* | *Hbp1^+/+^* | *Hbp1^-/-^* | *Hbp1^+/+^* | *Hbp1^-/-^* | *Hbp1^+/+^* | *Hbp1^-/-^* | *Hbp1^+/+^* | *Hbp1^-/-^* |
| Primordial follicle | 1441.4±209.56 | 1548.4±493.27 | 863.5±223.67 | 1031.29±308.67  (*) | 640.67±221.21 | 899.8±216.48  (*) | 254.67±59.02 | 411.83±204.9  (*) | 105.33±17.7 | 170.83±33.4  (**) | 14.17±5.7 | 81.67±55.4  (*) |
| Growing follicle | 352.25±121.74 | 389.67±145.48 | 381.67±45.21 | 574.13±113.97  (*) | 468.33±73.41 | 669±65.32  (*) | 149±57.05 | 213.67±49 | 186.83±22.8 | 306.33±82.4  (*) | 40.67±15.6 | 129±39.2  (**) |
| Total follicle | 1870.6±419.53 | 2093.6±615.08 | 1292±230.15 | 1683.57±303.94  (*) | 951±235.64 | 1568.8±183.93  (*) | 403.67±101.5 | 625.5±251.96  (*) | 292.17±31.9 | 477.17±111.4 (**) | 5.67±  19.4 | 210±94.0  (*) |

The data are presented as the means values ± SD. *p<0.5; **p<0.01.ß

Table S3 sgRNA of EGFP and the primers

| sgRNA | Sequence |
| --- | --- |
| EGFP sgRNA1  EGFP sgRNA 2 | GCGAGGAGCTGTTCACCG  AGCTGGACGGCGACGTAAA |
| EGFP sgRNA 3 | AGCGCACCATCTTCTTCA |
| EGFP sgRNA 4 | AAGTTCGAGGGCGACACCC |
| EGFP sgRNA 5 | TGAACCGCATCGAGCTGA |
| Pair1 （2,4） | AGCTGGACGGCGACGTAAA  AAGTTCGAGGGCGACACCC |
| Pair2 （1,5） | GCGAGGAGCTGTTCACCG TGAACCGCATCGAGCTGA |
| Pair3（2,3） | AGCTGGACGGCGACGTAAA  AGCGCACCATCTTCTTCA |
| EGFPN1-F1 | AAGGGCGAGGAGCTGTTCA |
| EGFPN1-R1 | TTGTGGCCGTTTACGTCG |
| EGFPN1-F2 | GGAGCGCACCATCTTCTTCA |
| EGFPN1-R2 | GCGGTTCACCAGGGTGTCGC |
| EGFPN1-F3 | CCCATCCTGGTCGAGCTGGACGG |
| EGFPN1-R3 | GCGGTTCACCAGGGTGTCGC |

Table S4 Primers for expression level of candidate genes in human KGN cells

| GAPDHF | TGTAGACCATGTAGTTGAGGTCA |
| --- | --- |
| GAPDHR | AGGTCGGTGTGAACGGATTTG |
| AXIN11F | GGTTTCCCCTTGGACCTCG |
| AXIN11R | CCGTCGAAGTCTCACCTTTAATG |
| ATMF | ATCTGCTGCCGTCAACTAGAA |
| ATMR | GATCTCGAATCAGGCGCTTAAA |
| ATRF | GGCCAAAGGCAGTTGTATTGA |
| ATRR | GTGAGTACCCCAAAAATAGCAGG |
| ARF1F | ATGGGGAACATCTTCGCCAAC |
| ARF1F | GTGGTCACGATCTCACCCAG |
| BLMF | TGTTACACCACCCCAAAGTCA |
| BLMR | GGAGGCAAATCAGTCTTTACTGT |
| BMPR1AF | TGAAATCAGACTCCGACCAGA |
| BMPR1AR | TGGCAAAGCAATGTCCATTAGTT |
| CHKAF | TGGTTCTGGAGAGCGTTATGT |
| CHKAR | CATTTTCTCGGCGATTTCTGC |
| CHKBF | GGCTTGCAGCAGTCTAAGTG |
| CHKBR | GGCACCATTGGTAGGCTCG |
| CDKN1CF | GCGGCGATCAAGAAGCTGT |
| CDKN1CR | GCTTGGCGAAGAAATCGGAGA |
| CTNNA1R | GGGGATAAAATTGCGAAGGAGA |
| CTNNA1R | GTTGCCTCGCTTCACAGAAGA |
| CTNNA2F | GGACGCTAACAGTGGAAAGG |
| CTNNA2R | GAGTGGCTTGCTCTACAGAGG |
| ETX1F | GCTCTTGTCTCGCCCTTTTGT |
| ETX1R | GTGGTGCAAGCCATTCCTAC |
| ETX2F | ATGTGTGCGTCGGTCAAGTAT |
| ETX2R | AGAATGGGGCCAAAACTGAAA |
| GPC3F | CCTTTGAAATTGTTGTTCGCCA |
| GPC3R | CCTGGGTTCATTAGCTGGGTA |
| HBP1F | TCATCACCATTGGAAGGAGGA |
| HBP1R | TTGCACCATCCCAAATCATCA |
| FBXW7F | CGACGCCGAATTACATCTGTC |
| FBXW7R | CGTTGAAACTGGGGTTCTATCA |
| NBNF | GACTGGCGTTGAGTACGTTGT |
| NBNR | TGATTTCGGCTGATCGACTGA |
| PTCH1F | CCAGAAAGTATATGCACTGGCA |
| PTCH1R | GTGCTCGTACATTTGCTTGGG |
| SMAD1F | AGAGACTTCTTGGGTGGAAACA |
| SMAD1R | ATGGTGACACAGTTACTCGGT |
| SMAD2F | CGTCCATCTTGCCATTCACG |
| SMAD2R | CTCAAGCTCATCTAATCGTCCTG |
| SMAD3F | TGGACGCAGGTTCTCCAAAC |
| SMAD3R | CCGGCTCGCAGTAGGTAAC |
| SMAD4F | CTCATGTGATCTATGCCCGTC |
| SMAD4R | AGGTGATACAACTCGTTCGTAGT |
| STK11F | TGTCGGTGGGTATGGACAC |
| STK11R | CCTTGCCGTAAGAGCCTTCC |
| TP53F | CAGCACATGACGGAGGTTGT |
| TP53R | TCATCCAAATACTCCACACGC |
| TSC1F | CAACAAGCAAATGTCGGGGAG |
| TSC1R | CATAGGGCCACGGTCAGAA |
| TSC2F | CCAAACCAACAAGCAAAGATTCA |
| TSC2R | CACATTCCATGCTCAGTTCTCT |
| WRNF | CACAGCAGCGGAAATGTCCT |
| WRNR | GAGCAATCACTAGCATCGTAACT |
| WT1F | CACAGCACAGGGTACGAGAG |
| WT1R | CAAGAGTCGGGGCTACTCCA |
| WISP1F | \| AGAGCCGCCTCTGCAACTT  TT \| \| --- \| \| GGA GAA GCC AAG CCC ATC A \| |
| WISP1R | GGAGAAGCCAAGCCCATCA |
| APCF | AAGCATGAAACCGGCTCACAT |
| APCR | CATTCGTGTAGTTGAACCCTGA |
| SPOPF | GAAATGGTGTTTGCGAGTAAACC |
| SPOPR | GCCCGAACTTCACTCTTTGGA |
| PTENF | TTTGAAGACCATAACCCACCAC |
| PTENR | ATTACACCAGTTCGTCCCTTTC |

Table S5 List of primers for evaluation of deletion efficacy

| HBP1F1 | TGATCCTACCCAATCTGG |
| --- | --- |
| HBP1R1 | attctgttagccagtcca |
| TP53F1 | atgatttgatgctgtccc |
| TP53R1 | aggagctgctggtgcagg |
| TP53F2 | ccagatgaagctcccaga |
| TP53R2 | GACGGAAACCGTAGCTGC |
| WT1F1 | cggtctatggctgccaca |
| WT1R1 | TAGGGCGTCCTCAGCAGC |
| WT1F2 | ttcgacgggacgcccagcta |
| WT1R2 | ccagcgagccctgctggcc |
| BRCA1F | gagcctacaagaaagtac |
| BRCA1R | gtgtcaagctgaaaagca |
| CTNNA1F | gccctctaataagaagag |
| CTNNA1R1 | TTGAGAAACTGGCTCTCC |
| CTNNA1F2 | gcagaagaacgttccgat |
| CTNNA1R2 | AAATGCCTGTGACCGCCT |
| CTNNA2F1 | atcattctgaaatgggac |
| CTNNA2R1 | ctgtgtaacaagtggctc |
| PTCH1F1 | ttgcggtggacaaacttcga |
| PTCH1R1 | ggggcggtccatgtaacc |
| NBNF1 | gaaccatacagacttttgac |
| NBNR1 | GACTGATCATTTTCAATC |
| NBNF2 | agtcaaacagatgaaatcc |
| NBNR2 | AACACTCCAAAAGTAATA |
| EXT1F1 | CCAGTGTTGAAGCTTCTCG |
| EXT1R1 | aaagcccccctccaaatt |
| EXT1F2 | gttatgagcagccgtttt |
| EXT1R2 | CCTCTGTTGTTGAAAGCA |
| TSC1F1 | cgtggcggaagtctatctcg |
| TSC1R1 | CGCAAAAAGGAGACGAAG |
| TSC2F1 | TGCACGATGGCCTTCAGCAGA |
| TSC2R1 | ccatcgtgcaggggcagggcg |
| SMAD3F1 | tcgtccatcctgcctttc |
| SMAD3R1 | gttctgctcgcccttctt |
| SMAD4F1 | actgttgcagatagcatc |
| SMAD4R1 | GTAAGTAGCTGGCTGACC |
| STK11F2 | atgttcacggagggcgagct |
| hSTK11R2 | tgccgtaagagccttccc |
| STK11F1 | ttcgcggcggacgacacct |
| STK11R1 | ctggacaccttctccggct |
| PTENF1 | ATCCAAACATTATTGCTATGGG |
| PTENR1 | ATCAATATTGTTCCTGTATACGC |
| WISP1F1 | GGGGTCAGCCTCATCACAGA |
| WISP1R1 | ATGGCAGCCTCCGTGCAGTT |
| WISP1F2 | CAACTGCACGGAGGCTGCCA |
| WISP1R2 | TTGCGTACCTCGGGCGGTC |
| FBXW7F1 | ggacctcagagcagccaa |
| FBXW7R1 | gctctgaaacatttttag |
| KRASF1 | acagcaggtcaagaggag |
| KRASR1 | catgtactggtccctcattg |
| XPAF1 | cggccgacggggctttgc |
| XPAR1 | cttccgctcgatactcgc |
| BMPR1AF1 | AACTAATGGACATTGCTTTG |
| BMPR1AR1 | cctgaagctaatgtggtt |
| WRNF1 | atgtggtgggatttgaca |
| WRNR1 | cacacaactgaattagtgca |
| SPOPF1 | tttgtgcaaggcaaagac |
| SPOPR1 | cgcagaagagggtaagct |
| GPC3F1 | AAGAAGGAGCGGACTTGGTGA |
| GPC3R1 | GATCTGTTGCAGCCGGAGCG |

Table S6 Primers for Real-time PCR

| Primers | Sequence |
| --- | --- |
| *Rpl19*F  *Rpl19*R | ATGAGTATGCTCAGGCTACAGA  GCATTGGCGATTTCATTGGTC |
| *Areg*F  *Areg*R | GGGGACTACGACTACTCAGAG  TCTTGGGCTTAATCACCTGTTC |
| *Ereg*F  *Ereg*R | TCCGAGGATAACTGTACCGC  CTCTCATGTCCACCAGGTAGAT |
| *Btc*F  *Btc*R | TGAAAACCCACTTCTCTCGGT  TGCTGGAGGTAAAACAGGTCC |
| *Amh*F  *Amh*R | GGCTAGGGGAGACTGGAGAA  CCAGAGTATAGCACTAACAGGC |
| *Gdf9*F  *Gdf9*R | CTGATAGGCGAGGTGAGACC  GAGCCGGACGGTATTGTAGA |
| *Zp3*F  *Zp3*R | GGTGTCCGTGGATACCGAC  GGCATCTTTCGTCATCTGCAC |
| *Oosp1*F  *Oosp1*R | CTCTGCTGAAGACGCAACTTA  AGGAGGATCACCAGTGAAATCTC |
| *Kitl*F  *Kit*R | CCCTGAAGACTCGGGCCTA  CAATTACAAGCGAAATGAGAGCC |
| *Npr2*F  *Npr2*R | GCTGACCCGGCAAGTTCTGT  ACAATACTCGGTGACAATGCAGAT |
| *Nppc*F  *Nppc*R | GGTCTGGGATGTTAGTGCAGCTA  TAAAAGCCACATTGCGTTGGA |
| *Hbp1*F  *Hbp1*R | TGGGAAGTGAAGACAAAT  TGACAGGGAGGACATACA |
| *Ppargc1a*F  *Ppargc1a*R | TATGGAGTGACATAGAGTGTGCT  CCACTTCAATCCACCCAGAAAG |
| *Ppargc1b*F  *Ppargc1b*R | TCCTGTAAAAGCCCGGAGTAT  GCTCTGGTAGGGGCAGTGA |
| *Nrf1*F  *Nrf1*R | AGCACGGAGTGACCCAAAC  TGTACGTGGCTACATGGACCT |
| *Nrf2*F  *Nrf2*R | TCTTGGAGTAAGTCGAGAAGTGT  GTTGAAACTGAGCGAAAAAGGC |
| *Tfam*F  *Tfam*R | ATTCCGAAGTGTTTTTCCAGCA  TCTGAAAGTTTTGCATCTGGGT |
| *Esrra*F  *Esrra*R | GGGGAGCATCGAGTACAGC  AGACGCACACCCTCCTTGA |
| *Mfn2*F  *Mfn2*R | AGAACTGGACCCGGTTACCA  CACTTCGCTGATACCCCTGA |
| CHIP *Tfam* P1F  P1R | CCCTGAGAAACTGGGGTATGGT  GATCCCGATGAAAAGAGGCAA |
| CHIP *Tfam* P2F1  P2R1 | AAAAAGACTTTGGAGAAGTG  TGCCAGACACATAGGAAAA |
| CHIP *Tfam* P2F2  P2R2 | TTTAGGTACCATGGAGCTGA  GGAAAACATGATACTCAATTCC |
