# Supplementary material for: Focused screening of mitochondrial metabolism reveals a crucial role for a tumor suppressor Hbp1 in ovarian reserve
Source: Cell Death Differ. 2016 May 20;23(10):1602–14. doi: 10.1038/cdd.2016.47 (PMC5041189; doi:10.1038/cdd.2016.47)
Supplement: Supplementary Information [file cdd201647x2.docx]

**Focused Screening of Mitochondrial Metabolism Reveals a Crucial Role for a Tumor Suppressor Hbp1 in Ovarian Reserve**

**Dong et al.**

**Supplementary Information**


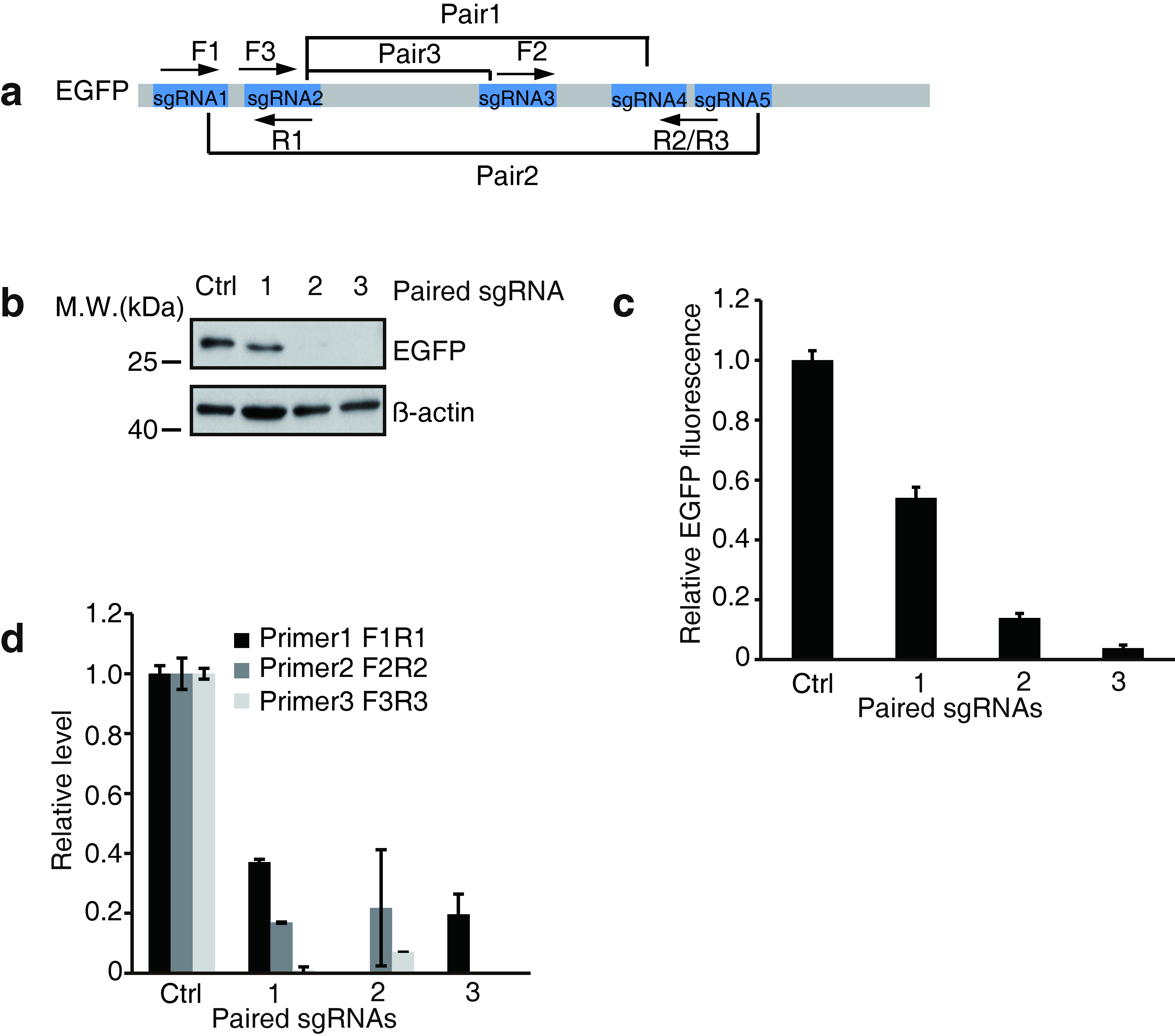


**Figure S1 Delivery of Cas9 and sgRNA Provides Efficient Depletion of EGFP in KGN-EGFP Cells**.

(a) Design strategy of the paired sgRNA and three primers for detecting EGFP deletion efficiency in KGN cells expressing EGFP (KGN-EGFP). Three paired sgRNAs are utilized in the following screen and the primers span the paired sgRNA DNA region.

(b) Western blot of EGFP level in KGN-EGFP cells transfected by Cas9 and three paired sgRNAs (paired sgRNAs: Pair1-3) vectors.

(c) EGFP fluorescence was detected by fluorescence microplate assay (Bio-Tek) as described as the instructions by Microplate Reader (Biotek, USA).

(d) Deletion efficiency by the optimized CRISPR system was determined through quantitative RT-PCR at the targeted region (Table S3).

All the data are presented as mean values ± SD (n = 3). The results are confirmed by 3 independent experiments.


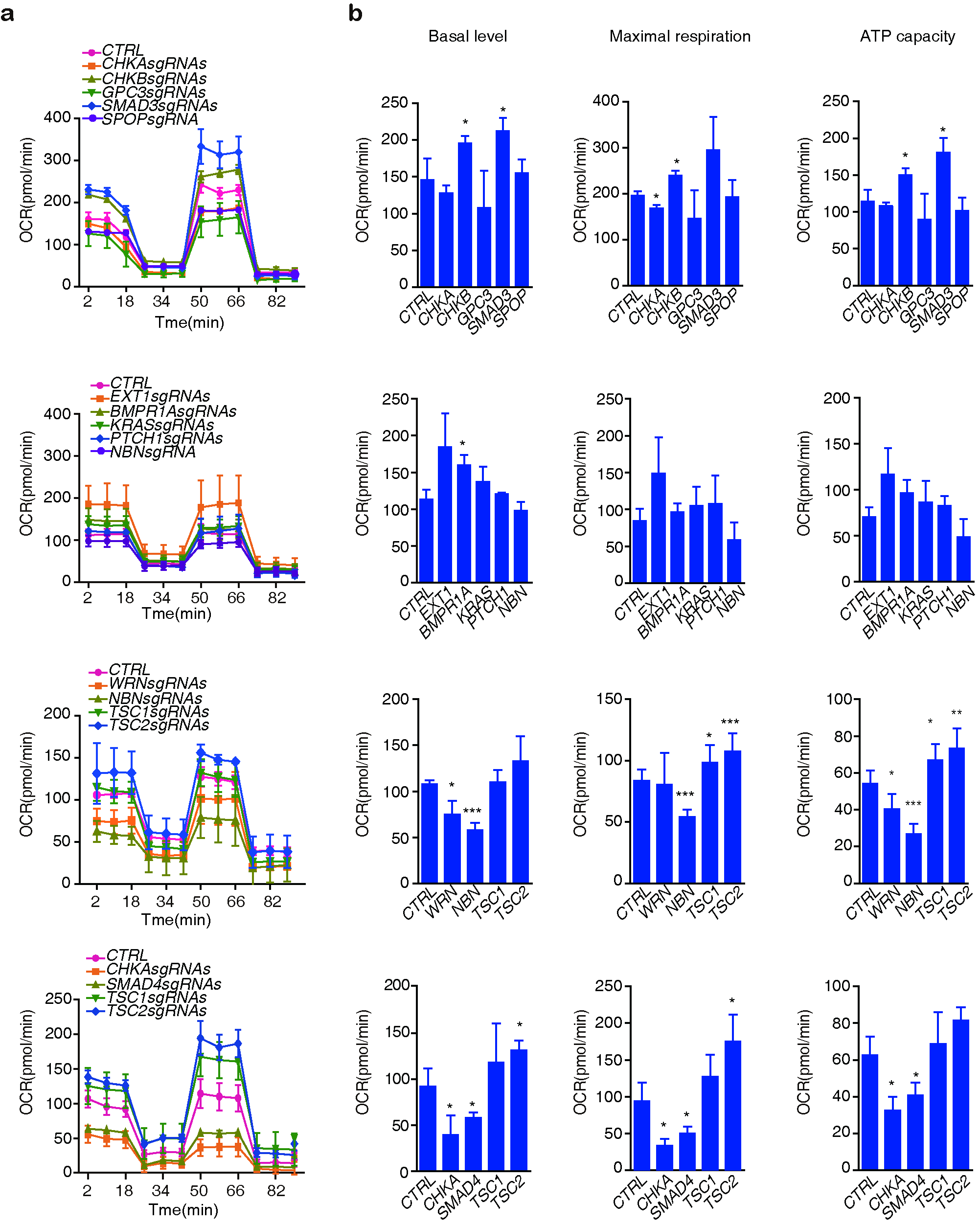


**Figure S2 Changes of Mitochondrial Bioenergetics after Deletion of Various Tumor Suppressor Genes in Human KGN Cells.**

(a) OCR (Oxygen Consumption Rate), indicative of OXPHOS, was monitored by using the Seahorse Bioscience Extracellular Flux Analyzer in real time. Cells were treated sequentially with oligomycin (Olig, 1 μM), p-trifluoromethoxy carbonyl cyanide phenyl hydrazone (FCCP, 0.5 μM), antimycin A (1 μM) and rotenone (Rote, 1 μM). Vertical lines indicate time points of the administration of corresponding inhibitors. The data are mean values ± SD (n = 3).

(b) Histograms of the basal respiration, maximal respiration and the ATP capacity of KGN cells deficient in various tumor suppressor genes compared to the normal controls. The data are mean values ± SD (n = 3). The results are confirmed by 3 independent experiments. * p < 0.05; ** p < 0.01.

**
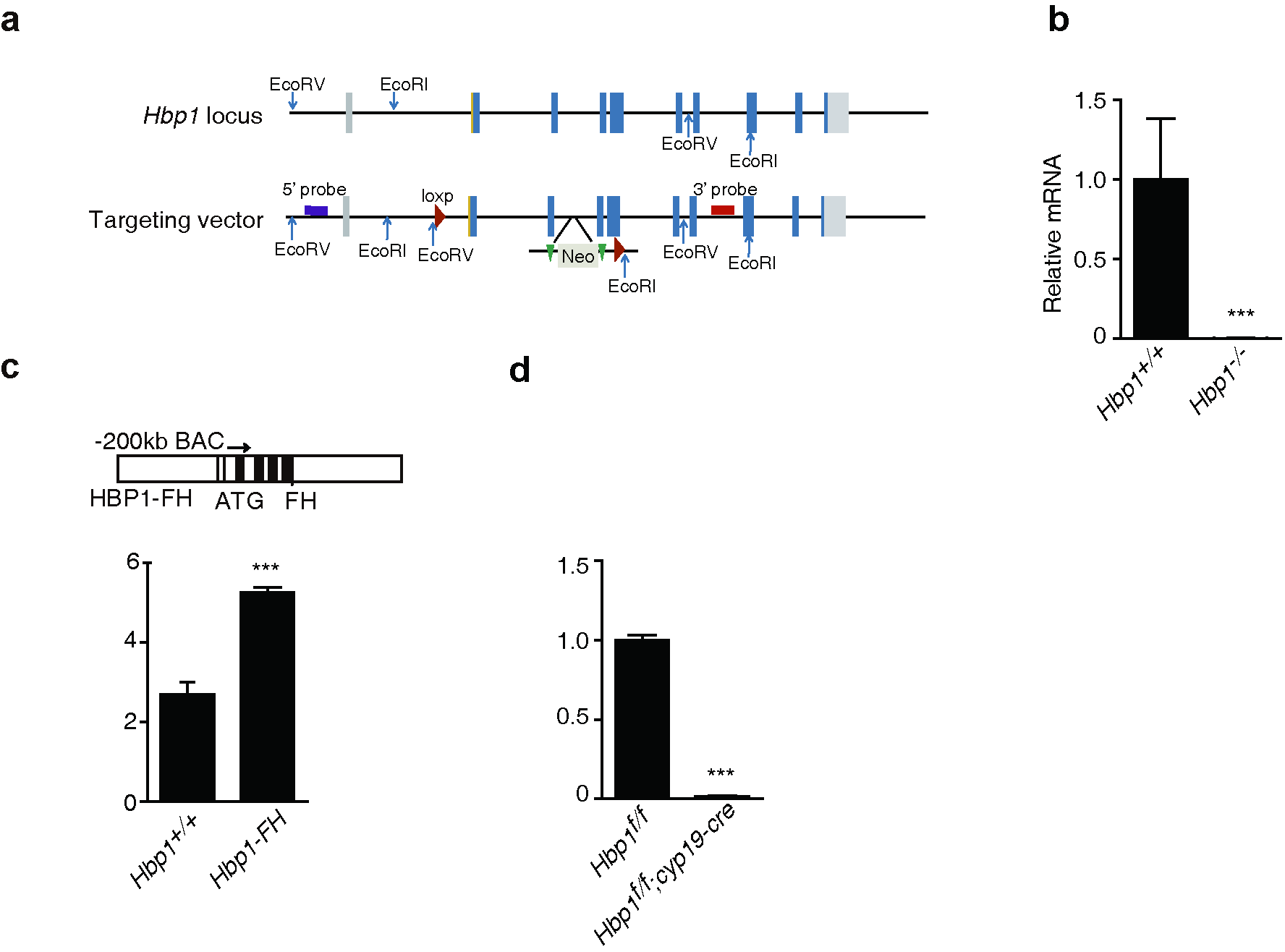
**

**Figure S3 Generation of Genome Engineered Mouse Models Including Conditional Knockout Mice and Transgenic Mice**

(a) Targeting strategy for the generation of a floxed *Hbp1* allele. One of the loxp element was insert into the intron between exon 1 and exon 2, and another loxp was inserted into intron between exon 3 and exon 4. Thus the exon 2 and exon 3 were deleted upon crossed with mice expressing *Cre* recombinase.

(b-d) Quantitative real-time PCR assay of *Hbp1* mRNA level in ovaries from these genetic engineered mice to evaluate the deletion efficiency.

All of the data are the mean values ± SD (n = 3), and the results are confirmed by 3 independent experiments. ** P < 0.01;*** P < 0.001

**
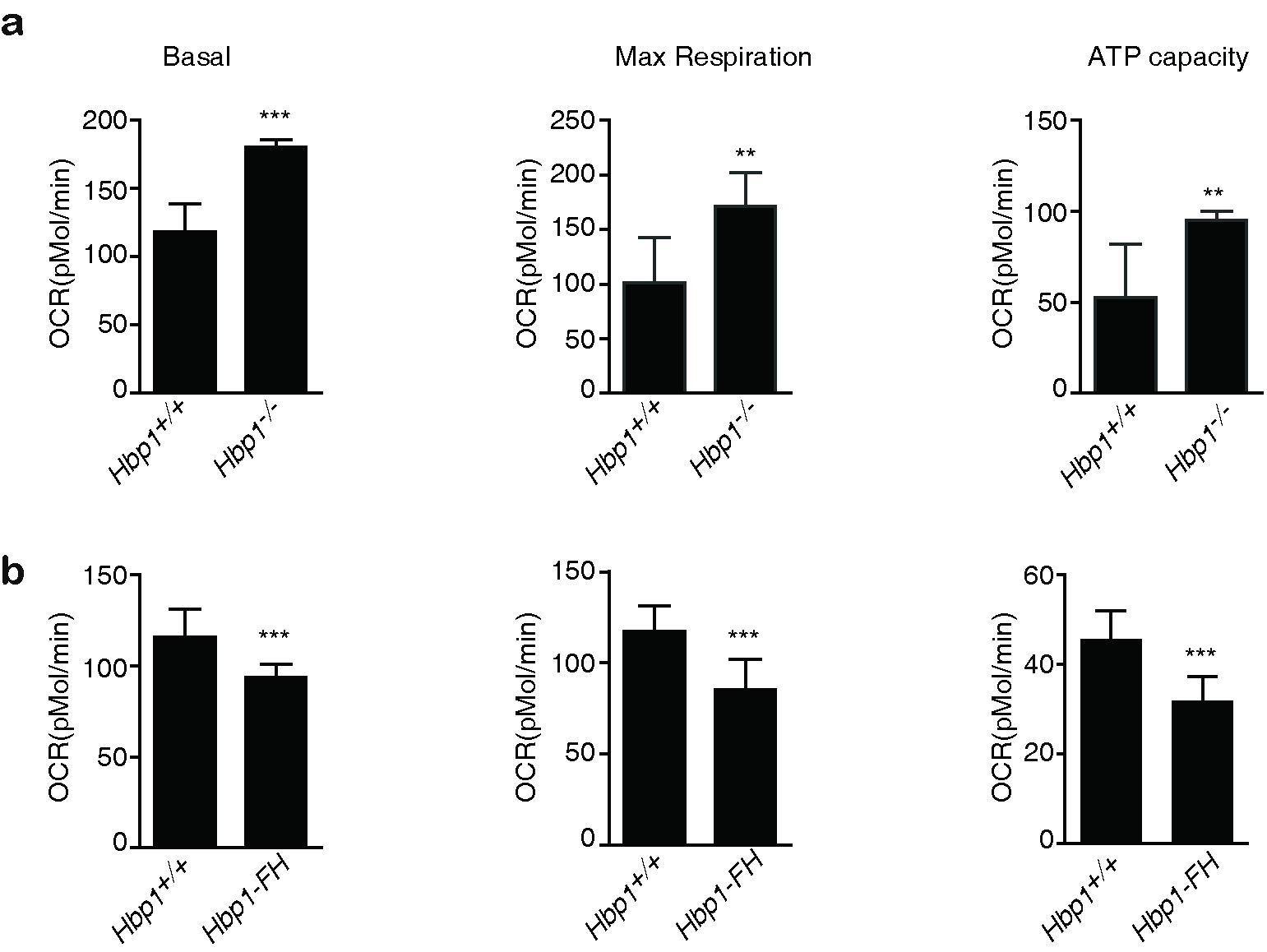
**

**Figure S4 Changes of Mitochondrial Metabolism, Biogenesis and Oxidative stress in Primary Ovarian Granulosa Cells with *Hbp1* Disruption.**

(a) Histograms of the basal respiration, maximal respiration and the ATP capacity of primary granulosa cells in *Hbp1^-/-^* and that in *Hbp1^+/+^* mice.

(b) Histograms of the basal respiration, maximal respiration and the ATP capacity of primary granulosa cells in *Hbp1-FH* and *Hbp1^+/+^* mice according to the OCR detected by Seahorse XF^e^24 in real time. The data are presented as the mean values ± SD (n = 3), and the results are confirmed by 3 independent experiments. ** P < 0.01;*** P < 0.001.

**
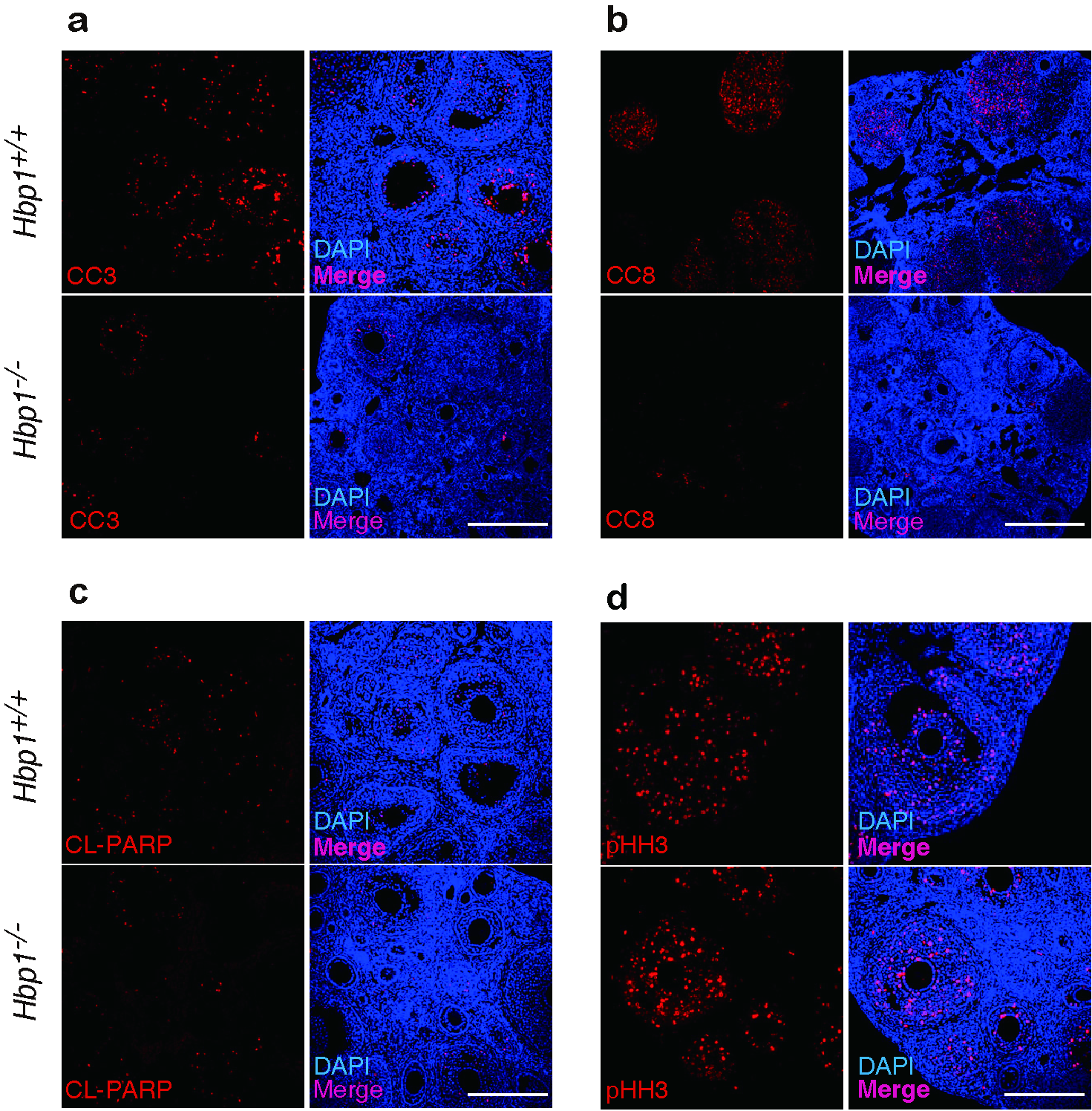
**

**Figure S5 Apoptosis of Granulosa Cells in Ovaries of Adult Female Mice.**

(a) Immunofluorescent stains for Cleaved Caspase 3 (CC3) in ovaries from 2-month-old mice. The blue indicated the nuclear stained with DAPI. The scale bar is 100 μm.

(b) Immunofluorescence of the Cleaved Caspase 8 (CC8) in *Hbp1^+/+^* and *Hbp1^-/-^* ovaries from 2-month-old mice. The blue indicated the nuclear stained with DAPI. The scale bar is 100 μm.

(c) Immunofluorescence of Cleaved PARP (Cl-PARP) in ovaries from 2-month-old females. The blue indicated the nuclear stained with DAPI. The scale bar is 100 μm.

(d) Immunofluorescence of Phosphorylated histone 3 (Ser10) (pHH3) in ovaries from 2-month-old females. The blue indicated the nuclear stained with DAPI. The scale bar is 50 μm. All the results are confirmed by 3 independent experiments.

**
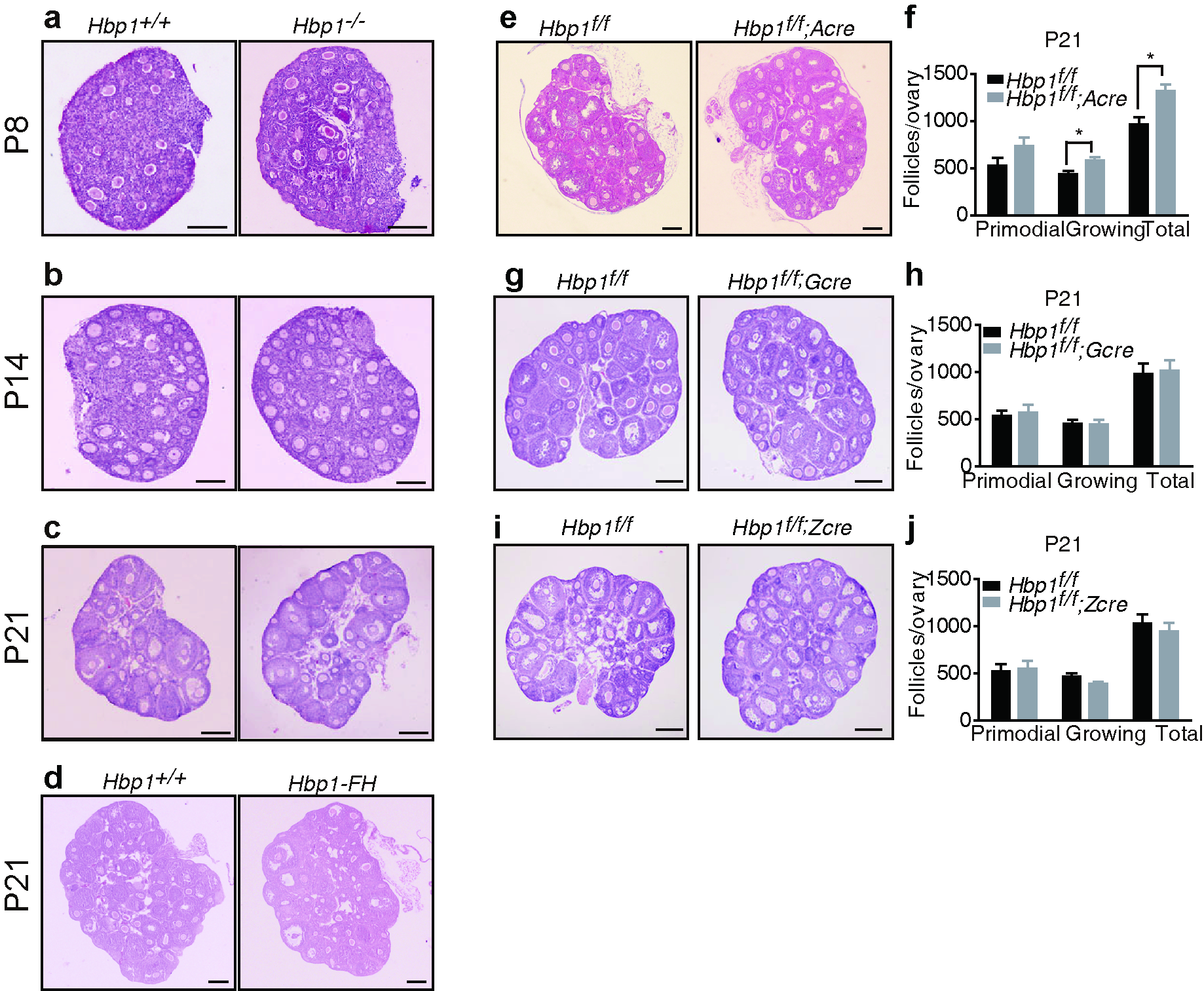
**

**Figure S6 Ovarian Morphologies of P8, P14 and P21 in mice**

(a-c) Ovaries from P8 (a), P14 (b) and P21 *Hbp1 ^-/-^* (c) mice and *Hbp1^+/+^* littermates were embedded in paraffin, and sections of 7 μm in thickness were prepared and stained with hematoxylin and eosin for observation.

(d) Ovarian morphology of WT and *Hbp1-FH* mice at P21.

(e, f) Ovarian morphology and follicle numbers of *Hbp1^f/f^;Amhr2-Cre* and *Hbp1^f/f^* mice at P21.

(g, h) Observation and quantification of ovarian follicles of *Hbp1^f/f^;Gdf9-iCre* and *Hbp1^f/f^* littermates at P21.

(i, j) Ovarian histology and follicle numbers of 21-day-old WT and *Hbp1^f/f^;Zp3-Cre* mice.

All the data are presented as the mean values ± SEM (n = 6). *p < 0.05. The scale bar is 100 μm.

**
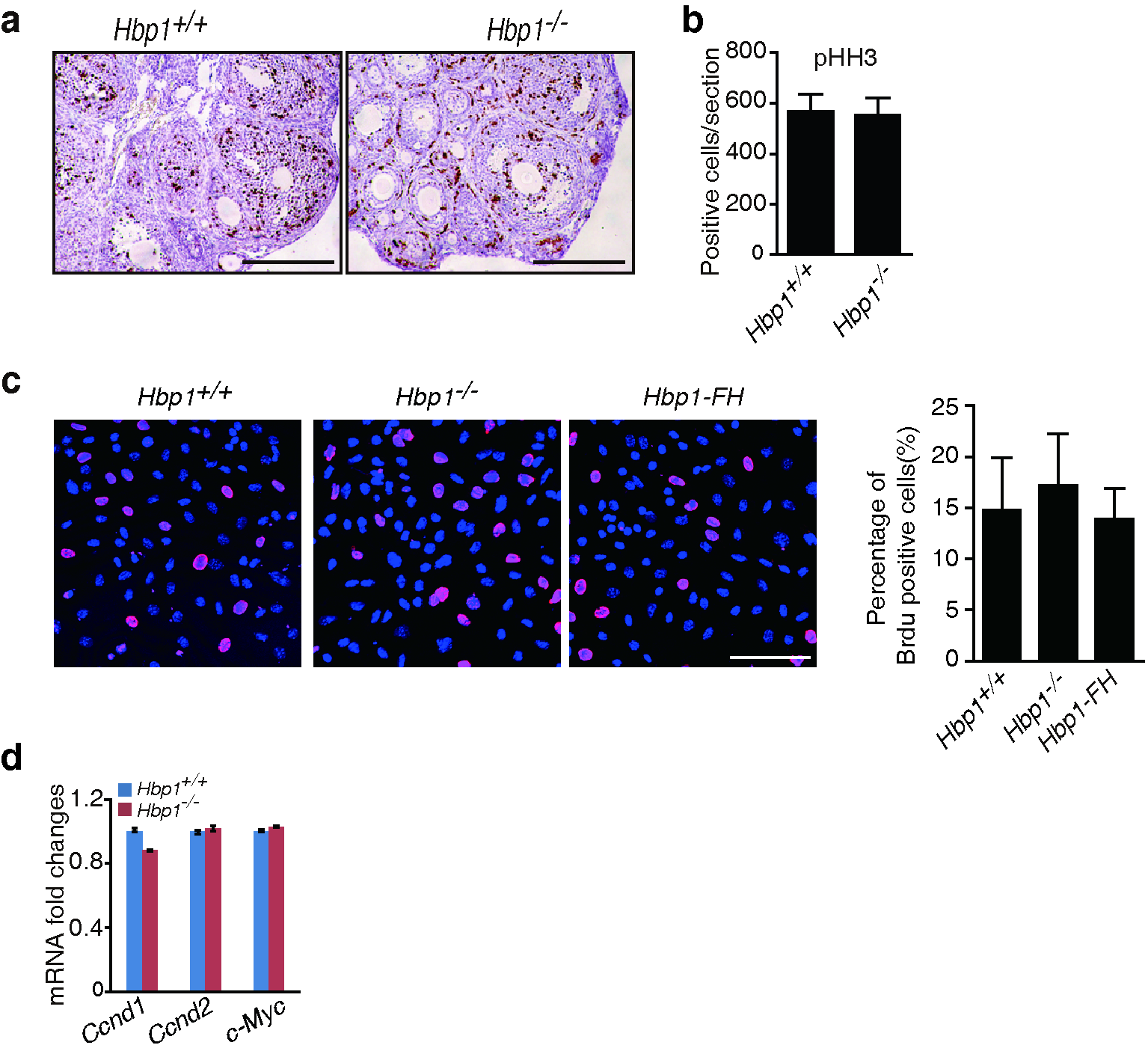
**

**Figure S7 Proliferation of Granulosa Cells After Ablation of *Hbp1***

(a) Immunohistochemical stains of phosphorylated histone 3 (Ser10) (pHH3) of sections from 21-day-old mice with PMSG treatment for 24 h. And the scale bar is 100 μm.

(b) Positive signals of pHH3 each section. Ovaries from mice treated with PMSG for 24 h were embedded in paraffin, and sections of 7 μm in thickness were prepared and processed for Immunohistochemical analysis with standard procedure in method. The positive signals in each section were observed under microscope. Each column represents the mean values ± SD (n = 4).

(c) Immunofluorescence staining of BrdU in primary GCs of indicated genotypes. Each column represents the mean values ± SD (n = 6). The scale bar is 50 μm.

(d) Expression of genes essential for granulosa cell proliferation, including *Ccnd1, Ccnd2, c-Myc*, in ovaries from mice treated with PMSG for 24 h.

Each column represents the mean values ± SD (n = 4).

**
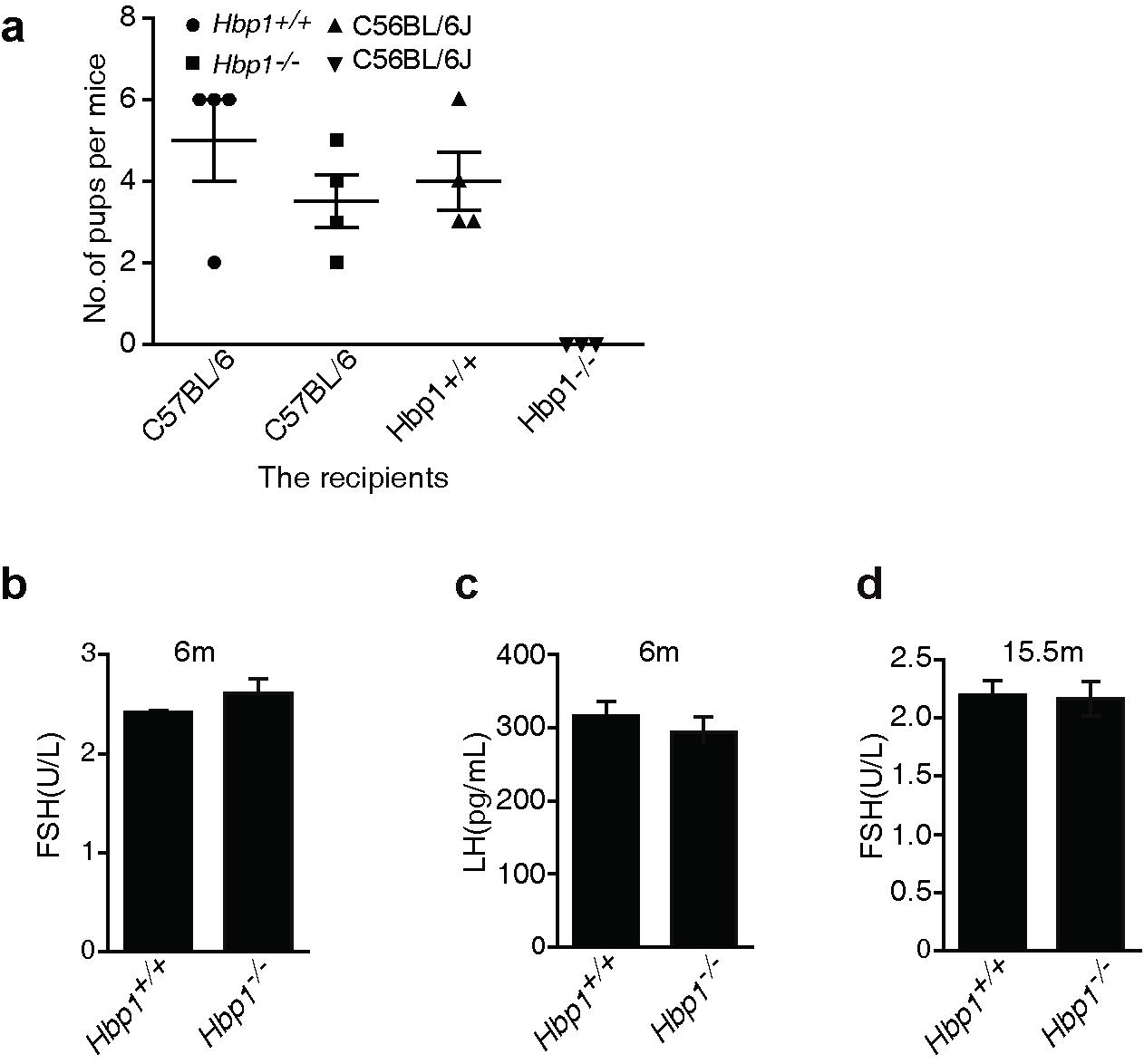
**

**Figure S8 Level of Follicle Stimulating Hormone (FSH) and Luteum Hormone (LH) in Serum from Old Mice**

(a) The number of pups from the recipients transplanted with ovaries from different donor females. The results are presented as means values ± SD (n = 3).

(b, c) Levels of serum FSH (b) and LH (b) from 6-month-old female mice. Each column represents the mean values ± SD (n = 4).

(d) Level of serum FSH from 15.5-month-old female mice. Each column represents the mean values ± SD (n = 4).
